# The role of ZIP transporters and group F bZIP transcription factors in the Zn‐deficiency response of wheat (*Triticum aestivum*)

**DOI:** 10.1111/tpj.13655

**Published:** 2017-09-17

**Authors:** Nicholas P. Evens, Peter Buchner, Lorraine E. Williams, Malcolm J. Hawkesford

**Affiliations:** ^1^ Rothamsted Research West Common Harpenden, Hertfordshire AL5 2JQ UK; ^2^ Biological Science University of Southampton Life Sciences Building 85, Highfield Campus Southampton SO17 1BJ UK

**Keywords:** zinc, micronutrient, biofortification, ZIP transporter, membrane transport, bZIP, wheat (*Triticum aestivum*), transcription factor

## Abstract

Understanding the molecular basis of zinc (Zn) uptake and transport in staple cereal crops is critical for improving both Zn content and tolerance to low‐Zn soils. This study demonstrates the importance of group F bZIP transcription factors and ZIP transporters in responses to Zn deficiency in wheat (*Triticum aestivum*). Seven group F TabZIP genes and 14 ZIPs with homeologs were identified in hexaploid wheat. Promoter analysis revealed the presence of Zn‐deficiency‐response elements (ZDREs) in a number of the ZIPs. Functional complementation of the *zrt1*/*zrt2* yeast mutant by TaZIP3, ‐6, ‐7, ‐9 and ‐13 supported an ability to transport Zn. Group F TabZIPs contain the group‐defining cysteine–histidine‐rich motifs, which are the predicted binding site of Zn^2+^ in the Zn‐deficiency response. Conservation of these motifs varied between the TabZIPs suggesting that individual TabZIPs may have specific roles in the wheat Zn‐homeostatic network. Increased expression in response to low Zn levels was observed for several of the wheat ZIPs and bZIPs; this varied temporally and spatially suggesting specific functions in the response mechanism. The ability of the group F TabZIPs to bind to specific ZDREs in the promoters of *TaZIP*s indicates a conserved mechanism in monocots and dicots in responding to Zn deficiency. In support of this, TabZIPF1‐7DL and TabZIPF4‐7AL afforded a strong level of rescue to the Arabidopsis hypersensitive *bzip19 bzip23* double mutant under Zn deficiency. These results provide a greater understanding of Zn‐homeostatic mechanisms in wheat, demonstrating an expanded repertoire of group F bZIP transcription factors, adding to the complexity of Zn homeostasis.

## Significance Statement

Understanding the molecular basis of Zn uptake, transport throughout the plant and homeostasis is an important step toward the improvement of both Zn content and tolerance to low Zn soils of the staple cereal, wheat. Here we identify and characterise Zn‐responsive members of the wheat ZIP membrane transporter family and also wheat F group bZIP transcription factors and determine their role in the response to Zn deficiency.

## Introduction

Micronutrient deficiency in humans is an issue of global concern. Enhancing the micronutrient content of staple crops is therefore an important objective for modern agriculture. Cereals such as wheat (*Triticum aestivum*) are relatively low in essential micronutrients such as zinc (Zn) and iron in their edible tissues. This presents a major problem when cereals form the main part of the diet and is a particular issue in developing countries (Kumssa *et al*., 2015). Crop yield is also detrimentally affected when plants are deficient in micronutrients as they are required throughout plant development (Brown *et al*., 1993). Zn has essential roles in plant growth, phytohormone activity, enzyme activation and modification of gene expression (Broadley *et al*., 2007). The yield reductions from reduced Zn availability are ultimately associated with damage to cell proteins, lipids and DNA (Cakmak, 2000). The development of crops that maintain growth and yield under low Zn availability would have clear benefits, and for that an understanding of the homeostatic network that determines Zn efficiency is required. Wheat cultivation occupies the largest area of any crop, therefore this study addresses mechanisms contributing to adaptation to low Zn in this monocot cereal.

Plants have developed sophisticated sensing and response mechanisms allowing them to adapt to variations in micronutrient availability. The complex nature of these homeostatic mechanisms is starting to be resolved in the model plant Arabidopsis. In this dicot, two bZIP (basic‐leucine zipper domain) transcription factors, bZIP19 and ‐23, are involved in adapting to Zn deficiency by inducing the expression of particular family members of membrane transporters, the ZIPs (ZRT, IRT‐related proteins) (Assunção *et al*., 2010). These have emerged as a key membrane transporter family in the journey of Zn from soil to seed (Palmgren *et al*., 2008). In Arabidopsis*,* eight members of the ZIP family have ability to transport Zn (AtIRT1, AtZIP1, ‐2, ‐3, ‐7, ‐10, ‐11 and ‐12) (Grotz *et al*., 1998; Milner *et al*., 2013), and several have been shown to be induced in response to Zn deficiency. *ZIP*s have also been characterised in cereals such as rice (*Oryza sativa*) and barley (*Hordeum vulgare*). In rice, OsZIP3, ‐4, ‐5 and ‐8 are functional Zn transporters (Ishimaru *et al*., 2005; Yang *et al*., 2009b; Lee *et al*., 2010a) while OsIRT1 and ‐2 as well as OsZIP6 and ‐7 can transport Fe but not Zn (Bughio *et al*., 2002; Ishimaru *et al*., 2006; Kavitha *et al*., 2015). The expression of *OsZIP4*,* ‐5* and ‐*8* is induced by Zn deficiency in both the root and shoot (Suzuki *et al*., 2012). In barley HvIRT1 and HvZIP3, ‐5 and ‐8 have all been shown to rescue the *zrt1*/*zrt2* Zn mutant yeast strain to a varying degree, indicating the ability to transport Zn (Pedas *et al*., 2008, 2009). Some of the rice and barley ZIPs are also induced by Zn deficiency, including *OsZIP1*,* ‐3*,* ‐4*,* ‐5* and *‐8* (Ramesh *et al*., 2003; Ishimaru *et al*., 2005; Lee *et al*., 2010b) and *HvZIP3*,* ‐5*,* ‐7*,* ‐8*,* ‐10* and *‐13* (Pedas *et al*., 2009; Tiong *et al*., 2013, 2015). In wheat little is known about this family except for *TdZIP1* from wild emmer wheat (*Triticum turgidum* ssp. *dicoccoides*), a Zn transporter with higher expression under Zn deficiency (Durmaz *et al*., 2011). In this study we have identified Zn‐responsive *ZIP*s in wheat (*T. aestivum*) and confirmed their Zn transport capability. A particular step forward was to investigate the mechanism that leads to changes in the expression of these transporters in response to Zn deficiency.

In Arabidopsis, increases observed in expression of particular ZIPs are proposed to occur via binding of AtbZIP19 and AtbZIP23 to Zn‐deficiency‐response elements (ZDREs) in ZIP promoters (Assunção *et al*., 2010). This operates as an adaptive mechanism to increase uptake and distribution of Zn in response to Zn deficiency. Evidence for this was provided by the extreme hypersensitivity of a *bzip19 bzip23* double mutant to low‐Zn conditions and the lack of Zn‐induced expression of key Zn transporters, including *AtZIP1*,* ‐3*,* ‐4*,* ‐5*,* ‐9* and *‐10* (Assunção *et al*., 2010). These genes contain one or more copies of the ZDRE in their promoter and direct binding by AtbZIP19 to this motif in *AtZIP4* was demonstrated previously (Assunção *et al*., 2010).

Further analysis of the *bzip19* and *bzip23* single mutants indicates that while they may overlap in their targets, *AtbZIP19* and *AtbZIP23* may also operate to alter the expression of a specific subset of genes (Inaba *et al*., 2015). The mechanism whereby low Zn is sensed is still to be elucidated, but it is suggested that under normal cellular Zn concentrations a Zn^2+^ ion binds to the cysteine–histidine‐rich motifs present in the group F bZIPs, making them inactive. Upon a reduction in cellular Zn concentration, Zn^2+^ dissociates, thereby activating the bZIP dimer which in turn binds to the ZDRE motif and brings about an increase in transcription of Zn‐responsive genes (Assunção *et al*., 2013). We show that wheat contains an additional level of complexity in adapting to Zn deficiency with an expanded number of group F bZIP transcription factors, allowing further modulation of the response to variations in Zn growth conditions.

## Results

### A multi‐gene family of ZIPs is present in wheat

**Figure 1:**
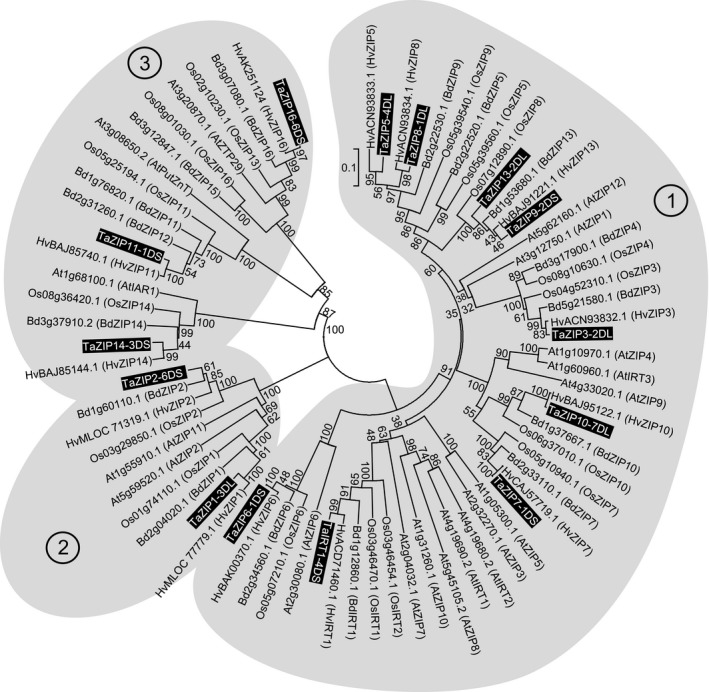
Phylogenetic tree of ZIP proteins from cereals and Arabidopsis. A neighbour‐joining tree was generated for Arabidopsis (At), rice, *Oryza sativa* (Os), *Brachypodium distachyon* (Bd), barley, *Hordeum vulgare* (Hv), and wheat, *Triticum aestivum* (Ta) *ZIP* coding sequence translations. The Muscle algorithm (Edgar, 2004) was used for the alignment of sequences and the phylogenetic tree was created using mega (v.5.2) software. Evolutionary distances were computed using the *p*‐distance method and are in the units of the number of amino acid differences per site. A thousand bootstrap replicates were used and bootstrap values are shown as percentages. Gene names of Arabidopsis, rice, *Brachypodium* and barley are shown in brackets, these gene names are in accordance with Tiong *et al*. (2015). D genome homeologs for *T. aestivum* are given in this figure. Full accession information for the wheat genes is provided in Table S2.

A comprehensive analysis of the genomic information recently available for hexaploid wheat was performed. From this analysis, 14 *TaZIP* genes were identified (Figure 1). Modern bread wheat (*T. aestivum*) is hexaploid, containing three genomes – A, B and D; homeologous genes are present on all three genomes. Full homeolog complements were found for all of the *TaZIP*s in each of the A, B and D genomes (Figure S1 in the Supporting Information). A previous phylogenetic analysis of *TaZIP*s identified 11 homologs (Tiong *et al*., 2015); *TaZIP2*,* ‐8* and *‐9* have not formerly been identified. The *TaZIP* homeologs group closely to one another (Figure S1) and for 12 of the *TaZIP*s the most closely related homolog was that from barley, followed by *Brachypodium* and rice. *TaZIP9* and *TaZIP13* showed high relatedness: *TaZIP9‐2DS* [*TaZIP9* present on the short arm (S) of chromosome 2D] and *TaZIP13‐2DL* [*TaZIP13* present on the long arm (L) of chromosome 2D] had 83% nucleotide sequence similarity (Table S1 and Figure S2), and both are located on chromosome 2, suggesting a wheat‐specific gene duplication. This is further supported by there being only one barley gene in this group.

The phylogeny of ZIPs displays three distinct clades (Figure 1). All clades contain both monocot and dicot ZIP homologs, suggesting that ZIPs were present before the divergence of monocots and dicots. *AtPutZnT*,* AtIAR1* and *AtZTP29* and their cereal homologs (*TaZIP11*,* TaZIP14* and *TaZIP16*) are situated in a subclade of clade 3 and are more distantly related to the other *ZIP*s included in the phylogenetic analysis. Each sub‐clade also contains monocot and dicot members, but there is also evidence suggesting species‐specific ZIP expansion with possible functional divergence.

### Wheat ZIPs functionally complement *zrt1/zrt2*, a Zn‐sensitive yeast mutant

**Figure 2:**
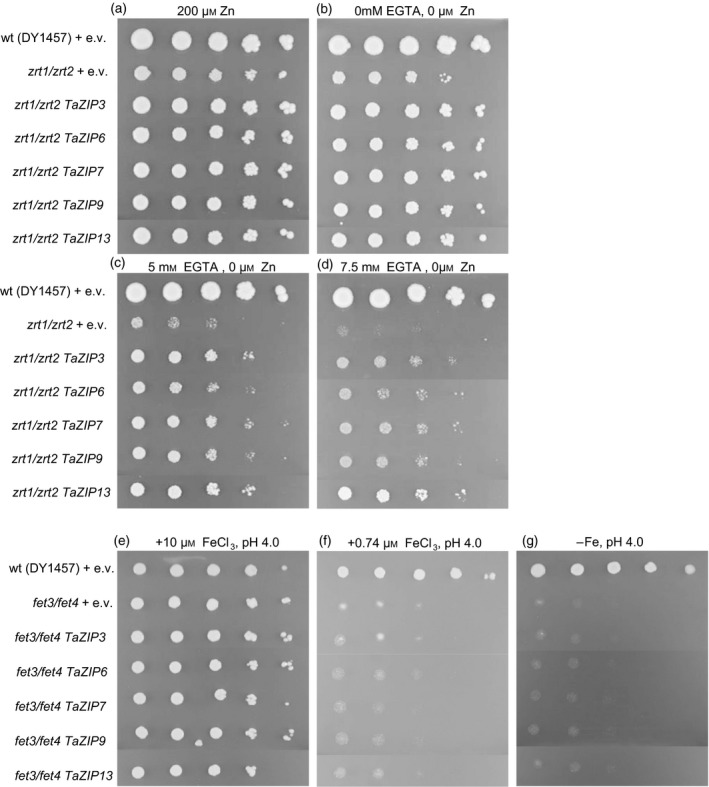
Complementation of Zn and Fe yeast uptake mutants with *TaZIP* genes. The yeast Zn uptake mutant *zrt1/zrt2* (a–d) and the Fe uptake mutant *fet3*/*fet4* (e–g) were transformed with five wheat *TaZIP* genes, shown here with empty vector controls (e.v.) in both the mutant and wild‐type yeast strains. Each spot is a dilution of the culture starting on the left of each plate (undiluted, 1:2, 1:10, 1:100, 1:1000) with the contents of selective media described above each plate.

A functional role in Zn homeostasis for a selection of the wheat ZIPs (*TaZIP3*,* ‐6*,* ‐7*,* ‐9* and *‐13*) was tested using yeast complementation. These TaZIPs were selected due to their location in the main clade of the ZIP phylogeny which contains previously confirmed Zn‐transporting ZIPs from Arabidopsis*,* rice and barley (Figure 1). These *TaZIP*s were tested using the *zrt1*/*zrt2* yeast mutant strain. This strain is defective in both the *ZRT1* high‐affinity and the *ZRT2* low‐affinity uptake transporters, and is susceptible to low‐Zn conditions (Zhao and Eide, 1996a,b). As seen in Figure 2, the vector‐transformed *zrt1*/*zrt2* yeast was markedly inhibited in growth compared with the wild type (DY1457) in the absence of Zn (achieved by addition of the chelator EGTA). Heterologous expression of *TaZIP3*,* ‐6*,* ‐7*,* ‐9* and *‐13* partially rescued the Zn‐deficiency phenotype. Growth levels in Zn‐deficient media (5 mm EGTA and 7.5 mm EGTA) were higher in the *zrt1*/*zrt2* strain expressing the *TaZIP*s compared with the empty pYES2 vector control, consistent with them functioning as Zn transporters. None of the *TaZIP*s heterologously expressed in the *fet3/fet4* Fe‐uptake mutant strain were able to rescue the Fe‐deficient phenotype (Figure 2e–g), suggesting that none of the *TaZIP*s investigated are functional Fe transporters.

### TaZIP expression is induced by Zn deficiency

**Figure 3:**
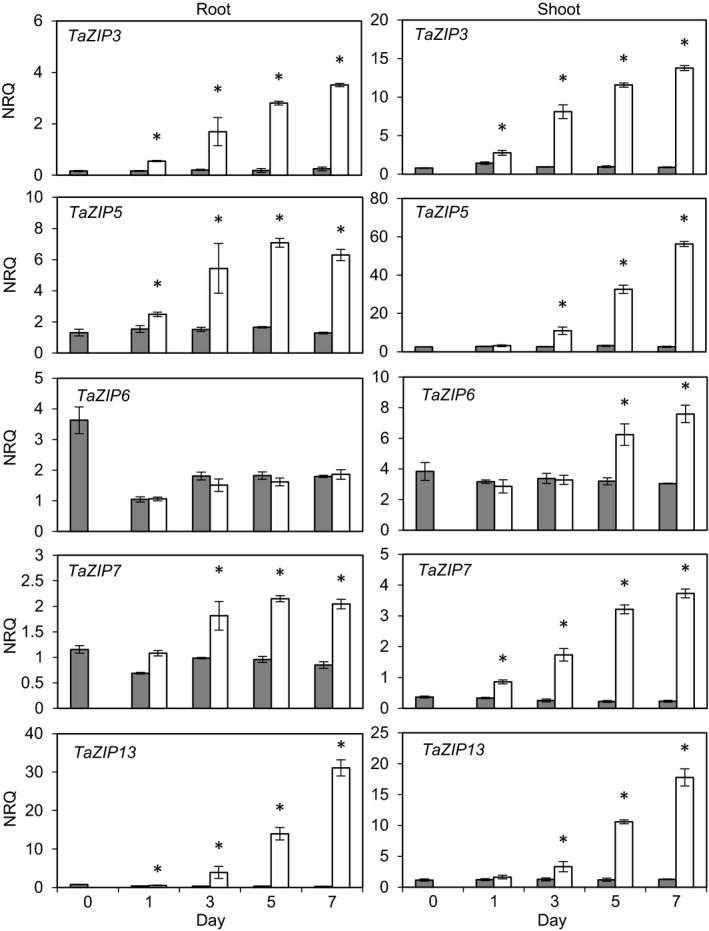
*TaZIP* gene expression analysis in wheat material throughout a 1‐week Zn starvation period. Relative expression levels (normalised relative quantification, NRQ) of five wheat *ZIP* transporter gene transcripts in root and shoot material throughout 1 week of Zn starvation. NRQ values are normalised to *TaActin3* and *TaSuccDH* expression and means of three biological replicates are given (±SEM). Bars within individual graphs displaying an asterisk show a significant difference between treatment means at a given time point. Significance (*P *< 0.05) was tested *post hoc*, using Fisher's least significant difference test on log_2_(1/NRQ)‐transformed data. +Zn = 8 μm Zn (grey bars), −Zn = 0 μm Zn (white bars).

Relative gene expression levels of *TaZIP3*,* ‐5*,* ‐6*,* ‐7* and *‐13* were determined in root and shoot tissue obtained during a 1‐week hydroponic Zn‐starvation period. Under these conditions there were no observable differences in wheat plants after 1 week and they showed similar root and shoot fresh weight at the end of this period (Figure S3). However, Zn levels were clearly reduced in both roots and shoots (Figure S3). Other elements were measured, but they showed little change over this period (Figure S4). There was a small but significant increase in root Mn levels in −Zn grown plants but no corresponding change in shoots (Figure S4). Thus the major effect was on the Zn content in both roots and shoots. Having established a system for testing the effects of Zn deficiency, we determined the transcriptional changes of a selected number of ZIPs. In roots, *TaZIP3*,* ‐5*,* ‐7* and *‐13* showed increased expression under the −Zn treatment, with only *TaZIP6* expression remaining fairly stable (Figure 3). *TaZIP3*,* ‐5* and *‐13* showed significantly higher expression levels from the first time point (day 1); *TaZIP7* expression was significantly increased from day 3. In the shoot material, expression of all five *TaZIP*s increased under −Zn conditions but the timing varied between individual genes. *TaZIP6* was the slowest to respond, remaining constant until day 5. As in the roots, *TaZIP3* showed significantly higher expression from day 1; however, *TaZIP5* and *‐13* were slower to respond in the shoot compared with the root. The magnitude of gene‐expression changes varied between the *TaZIP*s as well as between roots and shoots*. TaZIP5* expression increased to a greater extent in the shoots compared with roots, whereas for *TaZIP13* the opposite was observed. *TaZIP* induction under Zn‐deficient conditions was also observed over a longer starvation period (Figure S5). In this case, plants were only grown for 7 days rather than 14 days prior to the Zn starvation. Slight differences were observed, indicating that the developmental stage at which Zn starvation was implemented could have an effect, but overall the results were similar. In this case an induction was seen in *TaZIP6* expression in the root but again it was not as high as the induction observed for the other genes at day seven.

### Wheat contains seven group F *TabZIP*s with a full set of homeologs identified in the wheat genome

**Figure 4:**
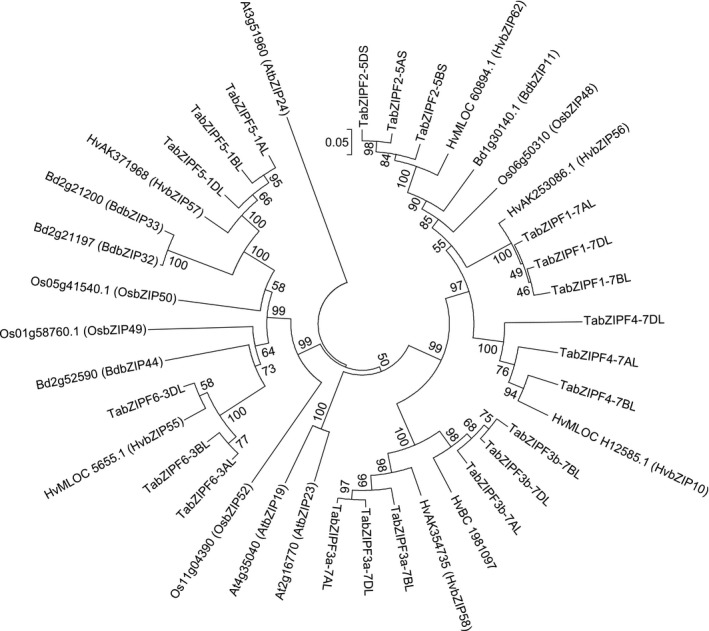
Phylogenetic tree of group F bZIP proteins from cereals and Arabidopsis. A neighbour‐joining tree was generated for Arabidopsis (At), rice, *Oryza sativa* (Os), *Brachypodium distachyon* (Bd), barley, *Hordeum vulgare* (Hv), and wheat, *Triticum aestivum* (Ta) group F *bZIP* coding sequence translations. The Muscle algorithm (Edgar, 2004) was used for the alignment of sequences and the phylogenetic tree was created using mega (v.5.2) software. Evolutionary distances were computed using the *p*‐distance method and are in units of the number of amino acid differences per site. A thousand bootstrap replicates were used and bootstrap values are shown as percentages. Gene nomenclature for Arabidopsis is from Jakoby *et al*. (2002), *Brachypodium* is from Liu and Chu (2015), rice is from Corrêa *et al*. (2008) and barley is from Pourabed *et al*. (2015). Full accession information for the wheat genes is provided in Table S2.

Searching the wheat genome revealed seven group F *TabZIP*s with homeologs identified on the A, B and D genomes. The previous genome‐wide identification of *TabZIP*s in the wheat genome (Li *et al*., 2015) reported 11 group F *TabZIP*s. However, this was inaccurate as closer examination showed that a considerable proportion of these are homeologs of the same gene (Table S2). Our analysis identified seven *TabZIPF*s, each with three homeologs. The phylogenetic analysis presented in Figure 4 shows that these have a related barley homolog. *TabZIPF3a* and *TabZIPF3b* are closely related to *TabZIPF3a‐7AL* and *TabZIPF3b‐7AL*, sharing 88% sequence similarity. Their sequence similarity, the location of each on chromosome seven and the fact that each has a barley homolog suggests they have evolved through gene duplication, which is likely to have occurred before the speciation of wheat and barley. *TabZIPF1, ‐2* and *‐4* also form a clade more closely related to *TabZIPF3a* and *‐3b* than *TabZIPF5* and *‐6. TabZIPF1*,* ‐2*,* ‐3a*,* ‐3b* and *‐4* cluster more closely to the two group F Arabidopsis *bZIP*s shown to be involved in the Zn‐deficiency response (*AtbZIP19* and *AtbZIP23*) rather than to *AtbZIP24,* which has a reported function in salt tolerance (Yang *et al*., 2009a).

### Group F bZIP expression is induced by low‐Zn conditions

**Figure 5:**
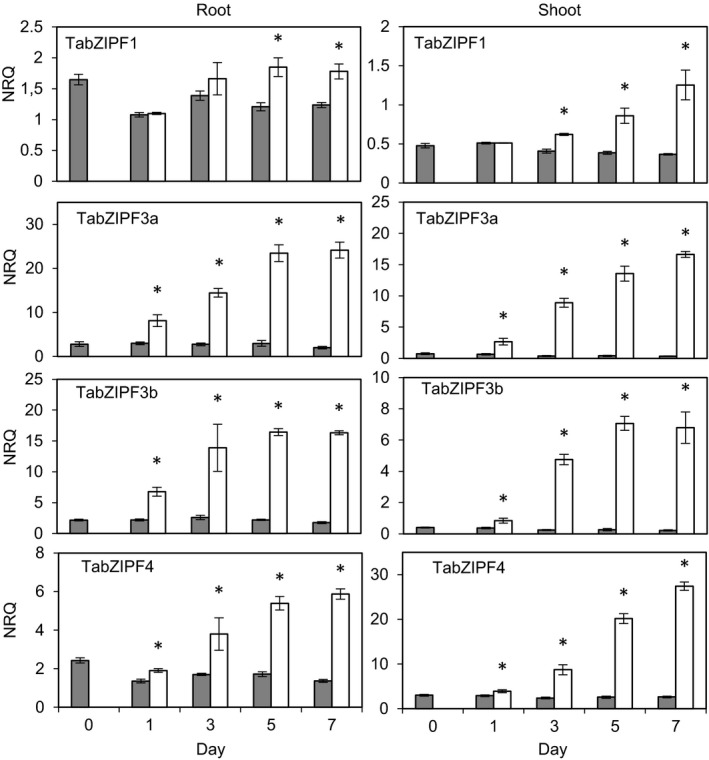
*TabZIP* gene expression analysis in wheat material throughout a 1‐week Zn starvation period. Relative expression levels (normalised relative quantification, NRQ) of five wheat *bZIP* transcription factor gene transcripts in root and shoot material throughout 1 week of Zn starvation. NRQ values are normalised to *TaActin3* and *TaSuccDH* expression and means of three biological replicates are given (±SEM). Bars within individual graphs displaying an asterisk show a significant difference between treatment means at a given time point. Significance (*P *< 0.05) was tested *post hoc*, using Fisher's least significant difference test on log_2_(1/NRQ)‐transformed data. +Zn = 8 μm Zn (grey bars), −Zn = 0 μm Zn (white bars).

The relative gene expression levels of *TabZIPF1*,* ‐3a*,* ‐3b* and *‐4* were determined in root and shoot material from a 1‐week period of Zn starvation. The four *TabZIPF*s were all induced under Zn‐deficient conditions in both roots and shoots but to rather different extents (Figure 5). *TabZIPF3a*,* ‐3b* and *‐4* showed the greatest induction in both roots and shoots. In contrast, *TabZIPF1* was slower to respond to the −Zn treatment, taking until day 5 in the root and day 3 in the shoot to be expressed to a significantly higher level. The magnitude of induction of *TabZIPF1* was less than that of the other *TabZIPF*s examined.

### Group F bZIPs rescue the Zn‐deficiency phenotype of an Arabidopsis *bzip19‐4 bzip23‐2* mutant indicating conserved homeostatic mechanisms

**Figure 6:**
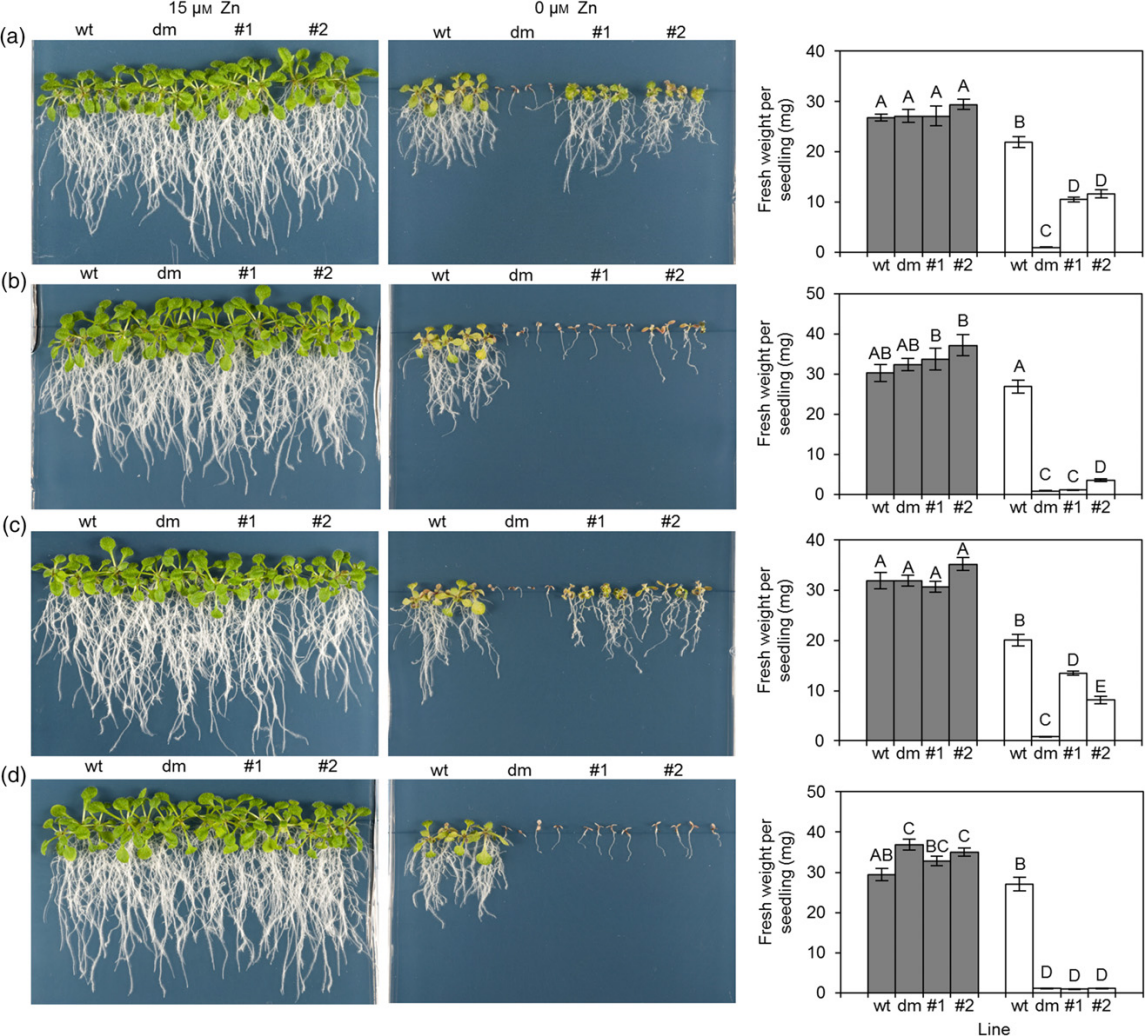
Functional complementation of the Arabidopsis *bzip19‐4 bzip23‐2* mutant with group F TabZIPs. Complementation analysis with (a) TabZIPF1‐7DL, (b) TabZIPF3b‐7BL, (c) TabZIPF4‐7AL and (d) TabZIPF4‐7DL. Fresh weight analysis results shown are mean average fresh weights per seedling ± SEM from six plates with four seedlings per plate, per line (*n *= 24). Bars with different letters indicate a significant difference (*P *< 0.05) tested on log‐transformed data using Fisher's least significant difference test. +Zn = 15 μm Zn (grey bars) and –Zn = 0 μm Zn (white bars). Representative plates at both the +Zn (15 μm Zn) and −Zn (0 μm Zn) media concentrations are shown. wt indicates the wild‐type line and dm indicates the untransformed *bzip19‐4 bzip23‐2* line. #1 and #2 are two corresponding, independent TabZIPF transformed *bzip19‐4 bzip23‐2* lines. All plates illustrated are 18 days post‐germination.

*TabZIPF1‐7DL*,* TabZIPF3b‐7BL*,* TabZIPF4‐7AL* and *TabZIPF4‐7DL* were investigated further due to variations in the conservation of their group F motif 1. They were cloned into the Arabidopsis expression vector pMDC32 (Curtis and Grossniklaus, 2003), which contains a dual 35S CaMV constitutive promoter. The sequences of the four cloned *TabZIPF*s are shown in Figure S6. All four cloned *TabZIP*s show high similarity to *AtbZIP19* and *AtbZIP23* in both the bZIP domain and group F motif 2; however, the group F motif 1 is less conserved. *TabZIPF3b‐7BL* and *TabZIPF4‐7DL* both contain a truncated group F motif 1 compared with the other group F *bZIP*s as well as their homeologs. *TabZIPF1‐7DL* and *TabZIPF4‐7AL* have a group F motif 1 with higher similarity to *AtbZIP19* and *AtbZIP23*. To test their importance in the Zn‐deficiency response mechanism, the ability of these four *TabZIP*s to rescue the Zn hypersensitivity of the Arabidopsis *bzip19 bzip23* double mutant was tested. An alternative double mutant Arabidopsis line, *bzip19‐4 bzip23‐2*, was createdby Nazri et al. (2017) that demonstrates the same Zn hypersensitivity as the Arabidopsis *bzip19‐1 bzip23‐1* double mutant previously characterised (Assunção *et al*., 2010). The *bzip19‐4 bzip23‐2* double mutant was transformed with the four *TabZIPF*s and two independent transgenic lines were characterised in detail. These were compared with the wild type and the non‐transformed double mutants on half MS media with (15 μm) and without (0 μm) Zn. Expression of both *TabZIPF1‐7DL* and *TabZIPF4‐7AL* significantly improved the performance of the *bzip19‐4 bzip23‐2* double mutant under low‐Zn conditions and plants displayed a significantly higher fresh weight than the mutant (Figures 6 and S7). *TabZIPF3b‐7BL* expression slightly increased the growth of the double mutant in one line only under low Zn, though to a lesser extent than both *TabZIPF1‐7DL* and *TabZIPF4‐7AL. TabZIPF4‐7DL* provided no rescue to the double mutant (Figure 6).

### Ability of TabZIP to bind to ZDRE motifs identified in TaZIP promoters

The rescue of the *bzip19 bzip23* double mutant by the wheat bZIPs suggested a conserved mechanism of action for these group F bZIP transcription factors. In Arabidopsis bZIP19 functions in binding to ZDRE motifs in Zn‐responsive genes (Assunção *et al*., 2010). Therefore, the presence of ZDREs in the promoters of *TaZIP* genes was determined. Regions upstream of the start codon were analysed at up to 2000 bp where possible. Motifs were regarded as potential ZDREs if they had no more than one mismatch to the ZDRE consensus (RTGTCGACAY) reported by Assunção *et al*. (2010). ZDREs were present in the promoter regions of a number of the ZIP genes; these are shown in Table S3 with their location relative to the predicted start codon. *TaZIP*s with a completely conserved ZDRE domain include *TaZIP5*,* ‐7*,* ‐8* and *‐10*; additionally *TaZIP1*,* ‐3*,* ‐6*,* ‐9* and *‐13* contain at least one ZDRE motif with one mismatch to the consensus in their promoter sequence.

**Figure 7:**
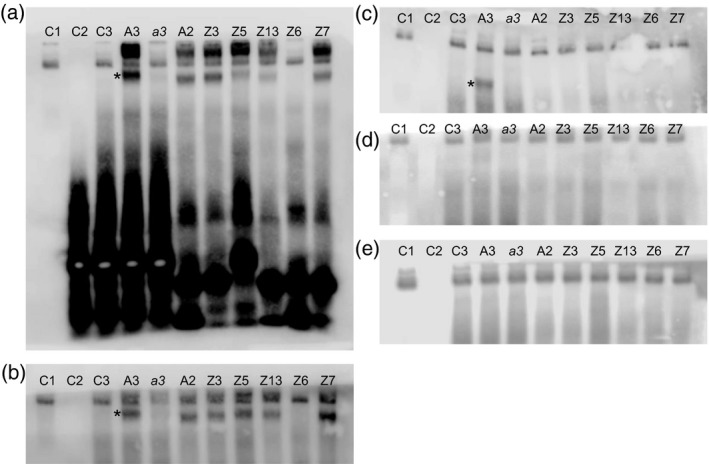
Electrophoretic mobility shift assays determining the binding ability of AtbZIP19 and TabZIPs to Zn‐deficiency‐response element (ZDRE)‐containing probes. Electrophoretic mobility shift assays were used to investigate the binding ability of AtbZIP19 (a) and five TabZIPs [TabZIPF1‐7DL (b), TabZIPF3b‐7BL (c), TabZIPF4‐7AL (d) and TabZIPF4‐7DL (e)] to Arabidopsis ZDRE‐containing probes and wheat ZDRE‐containing probes. Lanes C1–C3 are control lanes: C1 is TabZIPF protein only, no probe. C2 is probe (Ass3Z), no protein. C3 is probe (Ass3Z) with TNT wheat germ mix (no template). All other lanes contain *in vitro* synthesised TabZIPs (as labelled) with the following probes: lane A3 is Ass3Z, *a3* is Ass3Zmut (mutated Ass3Z) and A2 is Ass2Z which all contain Arabidopsis ZDREs. Z3–Z13 are TaZIP3ZDRE–TaZIP13ZDRE, which all contain wheat ZDREs. Asterisks indicate a band shift. Probe sequences are provided in Table S7.

To directly test the regulatory link between the *TabZIP*s and *TaZIP*s, the binding ability of *in vitro* synthesised bZIPs to putative ZDRE motifs discovered in the promoter regions of *TaZIP*s was determined (Figure 7). AtbZIP19, used as a control, created a band shift (indicative of binding) when two or three copies of the AtZIP4 ZDRE were present (probe names Ass2Z and Ass3Z), but not with a mutated three‐copy AtZIP4 ZDRE probe (Ass3Zmut). AtbZIP19 also produced band shifts with the previously untested TaZIP ZDRE probes 3, 5, 13 and 7. No shift was observed with the TaZIP6 ZDRE probe, suggesting AtbZIP19 does not interact with the putative ZDRE identified in the promoter of *TaZIP6* (Figure 7a).

TabZIPF1‐7DL created a band shift with Ass3z and Ass2Z, which are based on *AtZIP4* ZDREs, but not the mutated version, Ass3Zmut. TaZIP ZDRE probes 3, 5, 13 and 7 all produced a band shift indicating an interaction of TabZIPF1‐7DL with these ZDREs. As with AtbZIP19, however, TaZIP6 ZDRE did not produce a band shift (Figure 7b). TabZIPF3b‐7BL was only able to bind to the probe containing three AtZDREs (Ass3Z) and none of the TaZIP ZDRE probes (Figure 7c). Ass3Z is a modification of the ZDREs present in the promoter of *AtZIP4*. It has three ZDREs adjacent to each other whereas the promoter has two ZDREs spaced 128‐bp apart (Figure 7). Neither TabZIPF4‐7AL nor TabZIPF4‐7DL bound to any of the ZIP ZDRE probes tested (Figure 7d, e).

## Discussion

### TaZIPs have an important role in the transport of Zn

Analysis of the wheat genome revealed 14 *TaZIP*s, each with a homeolog on the A, B and D genomes. This in an increase in the number in the previous phylogenetic analysis of *TaZIP*s (Tiong *et al*., 2015) where 12 *TaZIP*s were identified and homeologs were not reported. The lengths ranged from 277 to 578 amino acids with much of the variation coming from the more divergent homologs *TaZIP11*,* ‐14* and *‐16* (homologs of *AtPutZnT*,* AtIAR* and *AtZTP29*, respectively). *TaZIP11*,* ‐14* and *‐16* were more distantly related to the other *TaZIP*s identified, suggesting a different role or cellular localisation in the plant. This is supported in knockout studies of *AtIAR* in Arabidopsis and heterologous expression of *AtZTP29* in yeast (Lasswell *et al*., 2000; Wang *et al*., 2010).

Functional characterisation using the yeast *zrt1*/*zrt2* mutant shows that *TaZIP3*,* ‐6*,* ‐7*,* ‐9* and *‐13* rescue the Zn hypersensitivity of this mutant. When considered alongside the Zn‐induced gene expression patterns of the *TaZIP*s determined in this study, this is strong evidence that ZIP members from this clade are important Zn transporters in wheat. Although not tested in yeast, *TaZIP5* was induced under Zn‐deficient conditions, suggesting a role in the Zn homeostatic framework. Barley homologs of three *TaZIP*s investigated in this study have shown Zn‐responsive expression (*TaZIP3*/*HvZIP3*,* TaZIP5*/*HvZIP5* and *TaZIP7*/*HvZIP7)* (Pedas *et al*., 2009; Tiong *et al*., 2015). Previously, from yeast studies, Pedas *et al*. (2009) indicated that *HvIRT1* and *HvZIP3, ‐5* and *‐8* could transport Zn. Additionally, work in yeast suggests that the wild emmer wheat (*T. turgidum* ssp. *dicoccoides*) *TdZIP1* (*TaZIP3* homolog) may also function as a Zn transporter (Durmaz *et al*., 2011). The five *TaZIP*s characterised in this study were able to complement the yeast *zrt1*/*zrt2* mutant but were unable to rescue the Fe‐uptake mutant *fet3*/*fet4*, suggesting there is substrate selectivity for these particular ZIPs.

The barley *TaZIP6* homolog *HvZIP6* is unresponsive to Zn status in the shoot but expression has been shown to increase in the root (Tiong *et al*., 2015). Additionally, *OsZIP6* expression is induced in both the roots and the shoots of Zn‐deficient rice plants (Kavitha *et al*., 2015). In this study we show that *TaZIP6* expression was not induced in the root during 1 week of Zn‐deficient growth but was significantly induced in the shoot after 5 days. The specific spatiotemporal expression patterns indicate that individual *TaZIP*s may have precise roles within the plant. Feasibly the Zn requirement of different regions in the root and shoot varies and may explain the differential pattern of increased gene expression initiation observed and its magnitude.

### Specific group F bZIPs induce the transcription of key Zn transporters, indicating a conserved homeostatic mechanism

The analysis presented here discovered seven distinct sets of group F bZIPs present in the wheat genome. Amongst the *TabZIPF*s identified (and between homeologs), conservation of the cysteine–histidine‐rich group F motif 1 varied. The group F motif 2 was widely conserved, as was the general bZIP domain. Previously, the two cysteine–histidine‐rich motifs present in group F bZIPs were hypothesised to be the binding site for Zn^2+^ ions (Assunção *et al*., 2010, 2013). As a small and efficient electron acceptor, Zn^2+^ is able to form tetrahedral complexes with sulphur and nitrogen ligands found in the side chains of cysteine and histidine (Tauris *et al*., 2009; Pace and Weerapana, 2014).

The role of four *TabZIPF*s in the Zn homeostatic mechanism was investigated by expression in the Arabidopsis *bzip19‐4 bzip23‐2* double mutant line. *TabZIPF1‐7DL* and *TabZIPF4‐7AL* provided partial complementation of this mutant, resulting in improved growth under the –Zn treatment compared with the untransformed *bzip19‐4 bzip23‐2* line. This indicates that the role of these genes in wheat is likely to be in the Zn‐deficiency response, and furthermore that they play a similar role in wheat and Arabidopsis. They did not completely rescue the *bzip19‐4 bzip23‐2* mutant. This may be due to slight differences in Zn^2+^ affinity of the TabZIPs, or a reduced binding ability to ZDREs in the promoter regions of Zn‐responsive Arabidopsis genes. TabZIPF1‐7DL and TabZIPF4‐7AL show divergence from AtbZIP19 and AtbZIP23 in the 3′ end of group F motif 1 (Figure S6), this may increase the Zn^2+^ affinity of these TabZIPs, although this requires further study.

Binding of AtbZIP19 and TabZIPF1‐7DL to ZDREs found in the promoter of *AtZIP4* as well as *TaZIP3*,* ‐5*,* ‐7* and *‐13* demonstrated that an analogous Zn‐homeostatic mechanism exists in the two species. *TabZIPF4‐7AL* partially rescued the *bzip19‐4 bzip23‐2* line, but did not bind to the ZDRE‐containing probes *in vitro*. This suggests this bZIP functions in the Zn‐deficiency response of wheat but the lack of binding indicates that either it acts through a different binding mechanism or that, potentially, binding is not observed here due to a requirement for native ZIP promoter flanking sequence or for a narrow and specific Zn concentration range not simulated in the electrophoretic mobility shift assay (EMSA).

*TabZIPF3b‐7BL* and *TabZIPF4‐7DL* were unable to restore the Zn‐deficiency response of the *bzip19‐4 bzip23‐2* mutant. Neither of these *TabZIPF*s contains a well‐conserved group F motif 1, which could be a causal factor for their lack of rescue ability. Despite being homeologs with high sequence similarity (84%), *TabZIPF4‐7AL* and *TabZIPF4‐7DL* showed different rescue abilities. This is most likely due to their different levels of group F motif 1 conservation. Neither TabZIPF3b‐7BL nor TabZIPF4‐7DL were able to bind to TaZDRE‐containing probes *in vitro*. Their presence in the wheat genome and expression *in planta* could be the result of genetic redundancy due to the hexaploidy of wheat, although an alternative hypothesis is a role in the fine tuning of the Zn homeostatic mechanism in wheat. The truncated motif in these *TabZIPF*s may facilitate the increased expression of specific Zn homeostatic genes at precise plant tissue locations. This may permit the plant to alter expression of particular genes in response to certain conditions or developmental stages without changing the expression of an entire suite of genes throughout the whole plant at a single given point in the Zn concentration continuum.

The expression profiles of *TabZIPF1*,* ‐3a*,* ‐3b* and *‐4* show they were induced, although to different degrees, under Zn‐deficient conditions. The current Zn‐homeostatic model for adapting to Zn deficiency suggests that dissociation of Zn^2+^ from group F bZIPs results in their binding to ZDRE motifs in the promoters of Zn‐responsive genes to increase their expression, seen particularly in the ZIPs (Assunção *et al*., 2010). Transcriptional regulation of the bZIPs themselves was not included as part of the model. Modifications may include a further level of intricacy, revealed here in wheat, whereby induction of bZIPs would allow for a sustained adaptive response under prolonged deficiency. Due to the differential effects observed across the *TabZIP*s in response to Zn deficiency, it is possible that certain *bZIP*s, notably the members with lower induction due to Zn deficiency, are ‘master regulators’ of other *bZIP*s*. TabZIPF1* may exert a global induction of *ZIP*s at critical levels of Zn deficiency while also influencing other bZIPs that then contribute to further adaptive responses. As such, *TabZIPF1* may be a promising target for future breeding or transgenic strategies.

In summary, our findings demonstrate that wheat contains a complex system for regulating Zn homeostasis. It has an expanded number of group F bZIP transcription factors that serve in altering the expression of ZIPs by binding to ZDREs in their promoters. These genes show differences in spatial and temporal expression patterns, indicating that there is a homeostatic network allowing adaptation to low Zn availability by contributing to Zn uptake and distribution under changing nutrient availability. The variation in conservation of cysteine–histidine‐rich motifs throughout the wheat group F bZIPs may provide additional refinement to the homeostatic mechanism.

## Experimental procedures

### Bioinformatics analysis

Gene and coding sequences of wheat *ZIP*s and *bZIP*s were identified using a Blast analysis of the IWGSC wheat survey sequence database and the TGACv1 assembly (http://www.plants.ensemble.org) with rice, barley and *Brachypodium* homologs. Phylogenetic analysis was performed by multiple protein sequence alignment using geneious v.8.1.3 (http://www.geneious.com; Kearse *et al*., 2012) with the Muscle algorithm (Edgar, 2004). mega5 (Tamura *et al*., 2011) was used for calculation of phylogenetic trees using the neighbour‐joining method with evolutionary distances computed using the *p*‐distance method and 1000 bootstrap replicates conducted for each phylogeny.

### Plant growth (*T. aestivum* and *Arabidopsis thaliana*)

#### Hydroponic culture 1‐week starvation experiment

*Triticum aestivum* cv. Paragon seedlings were germinated on sterile water‐soaked soft paper tissue for 7 days before transfer to single‐plant hydroponic aerated culture. Plants were cultivated for the first 3 days on half‐strength before changing to full‐strength modified Letcombe liquid nutrient solution [1.5 mm Ca(NO_3_)_2_, 5 mm KNO_3_, 2 mm NaNO_3_, 1 mm MgSO_4_, 0.5 mm KH_2_PO_4_, 25 μm FeEDTA, 0.2 μm CuCl_2_·2H_2_O, 1 μm H_3_BO_3_, 0.6 μm MnCl_2_.4H_2_O, 0.1 μm Na_2_MoO_4_·2H_2_O, 5 μm KCl, 8 μm ZnCl_2_, 2.56 mm 2‐(*N*‐morpholine)‐ethanesulphonic acid (MES) buffer, pH = 5.8; Drew and Saker, 1984] including the chelator 75.5 μm HEDTA. The solution was changed three times a week. Two weeks after germination, plants (apart from the +Zn control plants) were Zn‐starved by omitting the ZnCl_2_ from the culture solution (−Zn). Plants were grown in a growth chamber with 16‐h day conditions of 20°C/70% humidity and 500 μmol m^−2^ sec^−1^ light and night conditions of 16°C/80% humidity. Samples were taken at day 0, 1, 3, 5 and 7 of Zn starvation. Whole roots (washed with deionised water and dried briefly on soft paper towels) and whole shoots were sampled, weighed and immediately frozen in liquid nitrogen and stored at −80°C.

#### Hydroponic culture 3‐week starvation experiment

Conditions as above except for a reduction to 7 days rather than 14 days of hydroponic culture in +Zn solution prior to Zn‐starving the plants.

*Arabidopsis thaliana* plants were grown in growth chambers with 16‐h day conditions, 23°C and 120 μmol m^−2^ sec^−1^ light, night conditions 18°C. Soil used was autoclaved and comprised equal proportions of vermiculite, Levingtons M2 and John Innes No. 2 compost in 8‐cm pots supplemented with 0.28 g L^−1^ Intercept insecticide (Bayer, https://www.bayer.com/).

### Plant total RNA isolation

All wheat material was homogenised using a SPEX freezer mill (SPEX CertiPrep Ltd, http://www.spexcsp.com/) in liquid nitrogen, aliquotted into 2‐ml micro‐tubes and stored at −80°C. Total RNA was isolated by a modified method based on Verwoerd *et al*. (1989) with additional phenol–chloroform–isoamyl alcohol extractions. Possible genomic DNA contamination was removed by RNase‐free DNase treatment. The final air‐dried pellet was dissolved in an appropriate volume of RNase‐free water.

### Cloning full‐length TabZIPs and TaZIPs

First‐strand cDNA synthesis was performed from 2 μg total RNA and a dT‐adapter primer (Invitrogen Superscript III, http://www.invitrogen.com/; standard protocol, 2 h synthesis time). Full‐length TabZIPs were amplified using the oligonucleotide primers specified in Table S4 with *Pfu* DNA polymerase (Promega, http://www.promega.com/). TabZIP amplicons were subsequently topoisomerase‐cloned into pENTR/D‐TOPO (Invitrogen) and transformed into *Escherichia coli*. TabZIP plant expression vectors were created by Gateway (Invitrogen) recombination of pENTR:TabZIP vectors into pMDC32 (Curtis and Grossniklaus, 2003).

Full‐length TaZIPs were amplified using Q5 High‐fidelity DNA polymerase (New England Biolabs, https://www.neb.com/). TaZIP amplicons were cloned into pGEM‐T Easy vector (Promega) and subcloned into a pYES2 yeast expression vector using *Eco*RI digestion and subsequent ligation. All vectors were sequenced before Arabidopsis or yeast transformation.

### Functional complementation in yeast

The following strains of the yeast *Saccharomyces cerevisiae* were used in this study: wild‐type DY1457 (MATa, ade1/+ can1, his3, leu2, trp1, ura3), *zrt1*/*zrt2* (DY1457 +  zrt1::LEU2, zrt2::HIS3) and *fet3*/*fet4* (DY1457 +  fet3‐2::HIS3, fet3‐1::LEU2). Yeast strains were transformed with pYES2TaZIP vectors using the *S.c*. EasyComp Transformation Kit (Invitrogen) according to the manufacturer's instructions. Following transformation, PCR‐confirmed pYES2TaZIP containing *zrt1*/*zrt2* colonies was inoculated in 10 ml of SC‐glucose minus uracil (pH 5.3) overnight (30°C with shaking at 200 r.p.m.). Inoculums were pelleted (3 min, 1300 ***g***), suspended in SC‐galactose minus uracil (pH 5.3) and incubated for 4 h (30°C with shaking at 200 r.p.m.) to allow gene induction. Inoculums were then pelleted and washed with SC‐galactose minus uracil minus Zn (pH 5.3) to remove excess Zn from the pellet. Inoculums were diluted to OD_600_ = 0.4 using SC‐glucose minus uracil minus Zn (pH 5.3) and serial dilutions made (1:2, 1:10, 1:100 and 1:1000). Seven microlitres of the dilutions was plated onto SC‐galactose minus uracil plates (pH 5.3) containing either 200 μm ZnSO_4_ (+Zn), 0 Zn + 0 mm, 2 mm, 5 mm and 7.5 mm EGTA, incubated at 30°C and photographed after 8 days.

Following *fet3*/*fet4* transformation, positive colonies were confirmed using PCR and inoculated in 10 ml SC‐glucose minus uracil + 10 μm FeCl_3_ (pH 4.0) overnight (30°C with shaking at 200 r.p.m.). Inoculums were pelleted (3 min, 1300 ***g***), suspended in SC‐galactose minus uracil + 10 μm FeCl_3_ (pH 4.0) and incubated for 4 h (30°C with shaking at 200 r.p.m.) to allow gene induction. Inoculums were diluted to OD_600_ = 0.4 using SC‐glucose minus uracil + 10 μm FeCl_3_ (pH 4.0) and serial dilutions made (1:2, 1:10, 1:100 and 1:1000). Seven microlitres of the dilutions was plated onto SC‐galactose minus uracil minus Fe (pH 4.0) plates supplemented with 0 μm, 0.74 μm or 10 μm FeCl_3_, incubated at 30°C and photographed after 3 days.

### Expression analysis of TaZIPs and TabZIPs

Real‐time PCR was performed using the Applied Biosystems 7500 Real Time PCR System and the SYBR^®^ Green Jumpstart™ Taq ReadyMix™ (Sigma‐Aldrich, http://www.sigmaaldrich.com). The 25‐μl reactions contained 1 μl of cDNA and 250 nm of each primer. The primers used are given in Table S5 and were designed to cover gene expression of the homeologous genes from all three wheat genomes. Mean primer efficiencies were estimated using the linear phase of all individual reaction amplification curves (Ramakers *et al*., 2003) calculated using the LinRegPCR package (Ruijter *et al*., 2009). The stable, constitutive genes Actin 3 and Succinate Dehydrogenase were used to determine the normalised quantification of expression. The normalised relative quantity (NRQ) of expression was calculated in relation to the Ct values and the primer efficiency (*E*) of both the target gene (X) and the mean of the two normalisation reference genes (N) as normalised relative expression (NRE) based on Rieu and Powers (2009):

$$\mathrm{NRE}={\left(E_{X}\right)}^{-\mathrm{Ct},\;X}/{\left(E_{N}\right)}^{-\mathrm{Ct},\;N}$$

Statistically significant changes in relation to Zn treatment were calculated using a two‐way analysis of variance (anova) on log_2_(1/NRQ)‐transformed data followed by a *post hoc* Fisher's least significant difference test (LSD) at the 5% significance level. All statistical analyses were done in Genstat 17th edition (VSN International, https://www.vsni.co.uk/).

### Complementation of the Arabidopsis *bzip19‐4 bzip23‐2* mutant line with group F TabZIPs

pMDC32TabZIPF plasmids were transformed into *Agrobacterium tumefaciens* GV3850 by electroporation. *Arabidopsis thaliana* (Col‐8) *bzip19‐4 bzip23‐2* double mutants (Nazri *et al*., 2017) were transformed using the floral dip method (Clough and Bent, 1998) with an additional 3‐h pre‐induction of *vir* genes by the addition of 100 μm acetosyringone to the culture prior to floral dipping. Homozygous T_3_ plants were used in subsequent phenotype assays.

Growth assays were conducted using two independent T_3_ homozygous lines from each pMDC32‐TabZIPF transformation. Four sterilised seeds of each T_3_ line, the Col‐8 wild type and the untransformed *bzip19‐4 bzip23‐2* were plated onto six square plates of 0.5 ×  MS medium (Murashige and Skoog, 1962) containing 1% (w/v) sucrose and 0.8% (w/v) agarose containing 15 μm ZnSO_4_ (+Zn) and 0 μm ZnSO_4_ (−Zn). Following 18 days of growth, plates were photographed and root and shoot fresh weights were measured. Individual fresh weights were obtained by calculating the average from the combined weight of four seedlings prior to statistical analysis. A two‐way anova was performed on log‐transformed data for each complementation experiment (the four TabZIPFs investigated) followed by a *post hoc* Fisher's LSD at the 5% significance level.

### Electrophoretic mobility shift assay

Full‐length coding sequences of bZIPs were PCR‐amplified from sequenced vectors using primer pairs containing an SP6 promoter and Kozak region (forward) and a poly‐A tail (reverse) (Table S6). bZIP proteins were *in vitro* translated using the T_N_T SP6 High‐Yield Wheat Germ Protein Expression Kit (Promega) following the manufacturer's instructions with 690 ng of purified PCR product added to 18 μl of wheat germ master mix. ZDREs containing biotinylated oligonucleotides and nonbiotin‐labelled complementary oligonucleotides were synthesised (Table S7) (Eurofins, https://www.eurofins.com/) and annealed at a concentration of 1 pmol μl^−1^, 10 mm 2‐amino‐2‐(hydroxymethyl)‐1,3‐propanediol (TRIS), 1 mm EDTA, 50 mm NaCl for 5 min at 95°C and slowly cooling to room temperature (20°C) overnight. *In vitro* translated proteins (3 μl of the 30‐μl reaction) were incubated with 4 μl of the annealed oligonucleotide solution in a 20‐μl binding reaction containing 20 mm TRIS‐HCl (pH 7.5), 10 mm KCl, 1 mm EDTA, 0.25 μg μl^−1^ BSA, 1 mm DTT and 0.25 μg μl^−1^ salmon sperm DNA at 28°C for 30 min. The DNA–protein complex was analysed on a 6% native PAGE DNA retardation gel (Thermo Fisher Scientific, https://www.thermofisher.com/) using 0.5 × TRIS borate–EDTA at 100 V for 75 min. After electrophoresis, the gel was blotted to Amersham Hybond‐N^+^ membrane (GE Healthcare, http://www3.gehealthcare.com/en/global_gateway) using the XCell II™ Blot module (Thermo Fisher Scientific). The membrane was crosslinked using a Stratalinker^®^ UV crosslinker and the signal detected using Chemiluminescent Nucleic Acid Detection Module (Thermo Fisher Scientific) according to manufacturer's instructions using the Odyssey^®^ FC imaging system (Li‐Cor, https://www.licor.com/), with a 2‐min exposure.

### Determination of the Zn content in plant material

Cryogenically milled roots and shoots of hydroponically grown *T. aestivum* cv. Paragon were freeze dried for 2 days, then digested in 5 ml of nitric acid:perchloric acid (85:15, v/v; 70% concentration, trace analysis grade; Fisher Scientific) for a minimum of 2 h at room temperature followed by a 5‐h programmed thermoblock cycle. Five millilitres of 25% (v/v) nitric acid was added to the solution and the tubes were reheated for 1 h at 80°C. Ultra‐pure water (>18 MΩ) was added to to makel up to 9 ml, mixed well and re‐warmed for a further 30 min at 80°C. After cooling, the solutions were made up to final volumes of 10 ml with deionised H_2_O. Inductively coupled plasma optical emission spectrometry analysis was carried out using an Optima inductively coupled plasma–optical emission spectrometer (Perkin Elmer Life and Analytical Sciences, http://www.perkinelmer.com/).

## Conflicts of interest

The authors confirm that they have no conflicts of interest to declare.

## Supporting information


**Figure S1.** Phylogenetic tree of *Triticum aestivum* and barley ZIP proteins showing homeolog grouping.

**Figure** **S2.** Multiple sequence alignment of TaZIPs.

**Figure S3.** Phenotypic effect of Zn starvation throughout a 3‐week growth period.

**Figure S4.** Mineral concentration analysis of wheat root and shoot samples during a 1‐week Zn‐ starvation period.

**Figure S5.** Gene expression analysis of *TaZIP*s in wheat root and shoot material throughout an extended Zn starvation period of 3 weeks.

**Figure S6.** Multiple sequence alignment of cloned group F TabZIPs, AtbZIP19 and AtbZIP23.

**Figure S7.** Confirmatory PCR of 35S::TabZIP‐transformed Arabidopsis lines.

**Table S1.** TaZIP identity matrix**.**

**Table S2.** Wheat TaZIP and TabZIP gene identification details.

**Table S3.** Overview of ZDREs present in promoters of *TaZIPs*.

**Table S4.** Oligonucleotide primer sequences used for cloning of full length *TaZIPs* and *TabZIPs*.

**Table S5.** Oligonucleotide primer sequences used for SYBR Green real time RT‐PCR expression analysis.

**Table S6.** Oligonucleotide primer sequences used for PCR‐amplification of TabZIPs. With SP6 promoters and Poly‐A tails prior to in vitro transcription translation.

**Table S7.** Complementary oligonucleotides used in EMSAs.
