# Supplementary material for: The role of ZIP transporters and group F bZIP transcription factors in the Zn‐deficiency response of wheat (Triticum aestivum)
Source: Plant J. 2017 Sep 17;92(2):291–304. doi: 10.1111/tpj.13655 (PMC5656842; doi:10.1111/tpj.13655)
Supplement: Supplementary file 1 — Figure S1. Phylogenetic tree of Triticum aestivum and barley ZIP proteins showing homeolog grouping. [file TPJ-92-291-s001.pdf]

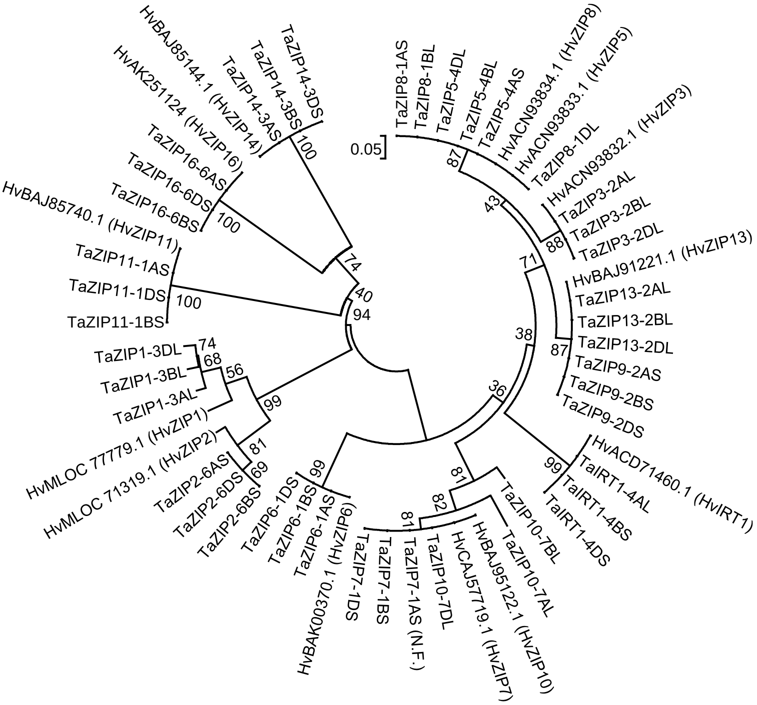

Figure S1. Phylogenetic tree of *T. aestivum* and barley ZIP proteins showing homeolog grouping. A neighbour joining tree was generated for barley *Hordeum vulgare* (Hv) and wheat *T. aestivum* (Ta) *ZIP* coding sequence translations. The Muscle algorithm (Edgar, 2004) was used for the alignment of sequences and the phylogenetic tree was created using MEGA (v5.2) software. Evolutionary distances were computed using the p-distance method and are in the units of the number of amino acid differences per site. 1000 bootstrap replicates were used and bootstrap values are shown as percentages. All homeologs for *T. aestivum* are given in this figure.
