# Supplementary material for: The role of ZIP transporters and group F bZIP transcription factors in the Zn‐deficiency response of wheat (Triticum aestivum)
Source: Plant J. 2017 Sep 17;92(2):291–304. doi: 10.1111/tpj.13655 (PMC5656842; doi:10.1111/tpj.13655)
Supplement: Supplementary file 2 — Figure S2. Multiple sequence alignment of TaZIPs. [file TPJ-92-291-s002.pdf]

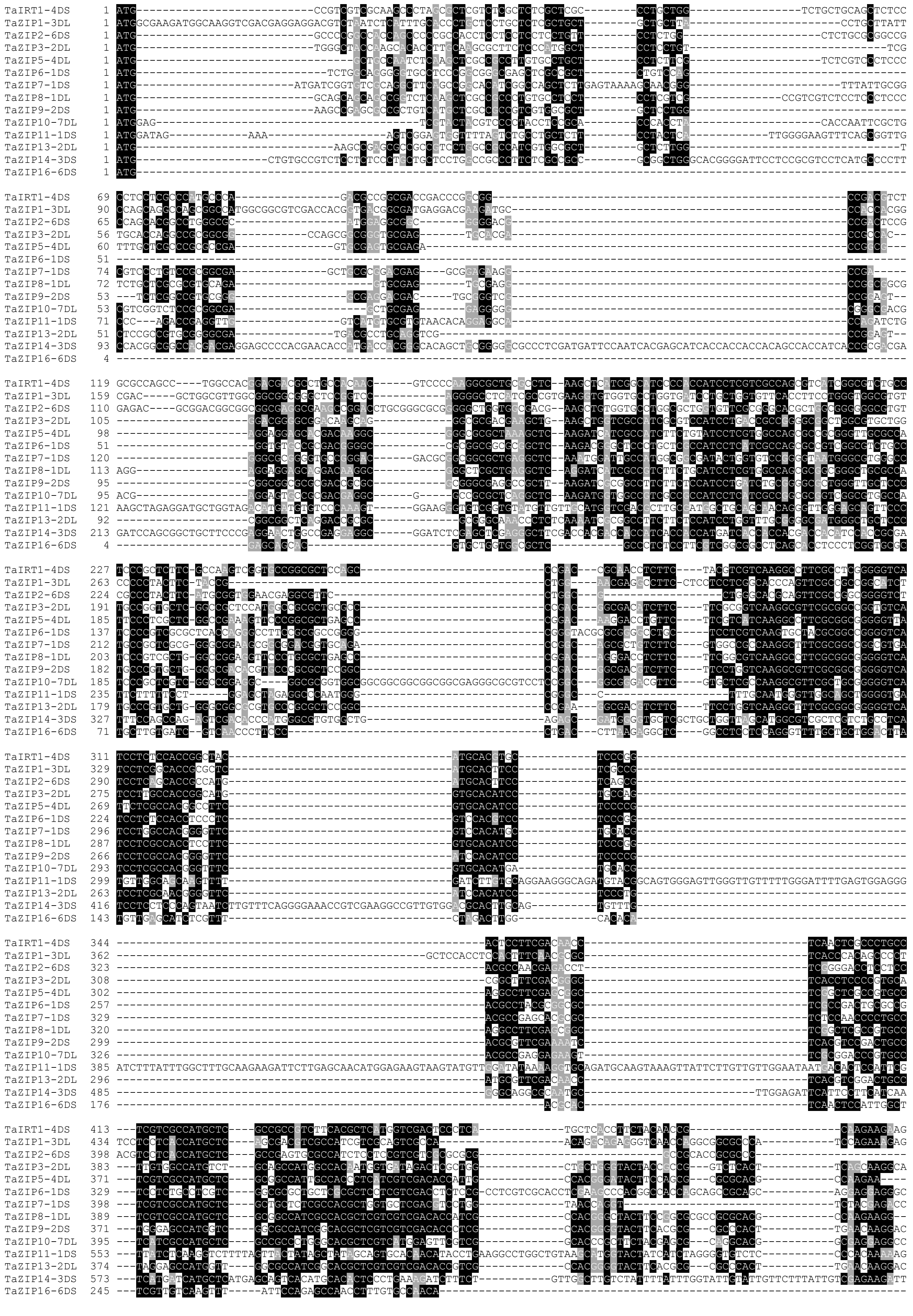

Figure S2. Multiple sequence alignment of TaZIPs. The Muscle algorithm (Edgar, 2004) was used for the alignment of sequences and the BoxShade software was used to generate this figure.

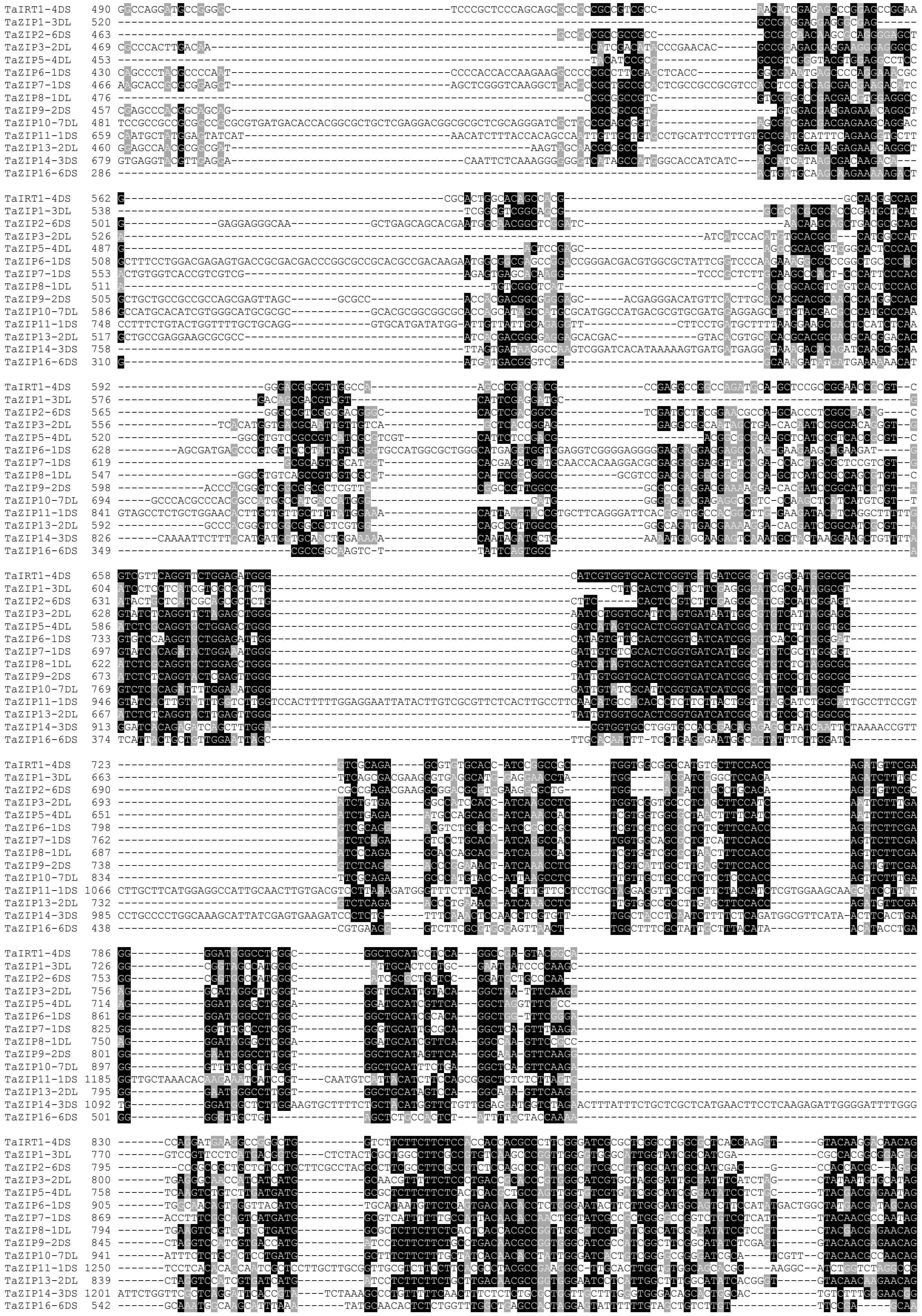
TaIRT1-4DS
TaZIP1-3DL
TaZIP2-6DS
TaZIP3-2DL
TaZIP5-4DL
TaZIP6-1DS
TaZIP7-1DS
TaZIP8-1DL
TaZIP9-2DS
TaZIP10-7DL
TaZIP11-1DS
TaZIP13-2DL
TaZIP14-3DS
TaZIP16-6DS

```
TaIRT1-4DS   927 CCCCACCG------CGCTCATCGTCGTCGGCCTGCTCAACGCCGCCTCCGCGGGGCT--GCTGCACTA----------------------------------------------------
TaZIP1-3DL   867 CCCGGCCG------CGGACTGGACATATGCCATCTCCATGGGGATCGCCACGGGGGT--CTTCGTCTA----------------------------------------------------
TaZIP2-6DS   894 CGCCGTGG------CCGACTGGATCTTCGCCGTCTCCATGGGCCTCGCCACCGGCAT--CTTCATCTA----------------------------------------------------
TaZIP3-2DL   897 CTCTACTG------CCTTCATTATTGAGGGAGTCTTCAACTCAGCCTCGGCAGGGAT--TTTGATCTA----------------------------------------------------
TaZIP5-4DL   855 CCCTAGGG------CCCTAATTGTGGAAGGGCTCCTCAGCGCTGCCGCCGCCGGAAT--TCTGAACTA----------------------------------------------------
TaZIP6-1DS  1008 TCCAAACG------CCCTAATAATTGAAGGCCTTCTTGGTTCACTCTCTGCCGGAAT--CCTTGTCTA----------------------------------------------------
TaZIP7-1DS   966 CCCCAGGG------CTCTAGTGGTTGAAGGCATCCTCGATTCTGTGTCGGCCGGCAT--ACTCATATA----------------------------------------------------
TaZIP8-1DL   891 CCCCAACA------CCCTGATCATCCAAGGGATCCTCAGCGCCGCCGCCGCCGGAAT--CCTCAACTA----------------------------------------------------
TaZIP9-2DS   942 CCCGACGG------CGCTGGTGGTGGAGGGCAGCCTCAACTCTGTGGCAGCGGGGAT--CCTCGTCTA----------------------------------------------------
TaZIP10-7DL 1038 CCCCAGGG------CATTGGTGGTGGAAGGCATTCTGGATTCAATGTCTTCTGGTAT--ACTGATCTA----------------------------------------------------
TaZIP11-1DS 1356 CTACATGGTTCTTCCAGTCTCTCTTCACGGCCTACCACGGGGTGCTGCTGTTGCTAG--TTGTGTATATGGTGCCACTGATAGCTGGCGAGGAGCCTTGGCAGCTGCAGCTCTGACCGGG
TaZIP13-2DL  936 CCCGACGG------CGCTGGTGGTGGAGGGCAGCCTCAACTCCGTGGCAGCGGGGAT--CCTCATCTA----------------------------------------------------
TaZIP14-3DS 1307 ATCCAGGA------CATTCCTCTTTGA--------TTGAGGGATTTACTGCTGGTGG--TTTTATTTA----------------------------------------------------
TaZIP16-6DS  630 CTTAAATC------CTGAAATTCTTGAAGGCCTACTTGCATCAGTTGGTGGTGTTATGGCTTTCCTCA----------------------------------------------------

TaIRT1-4DS   987 --------------CATGGCGCTCGTCGAG-CTCCTCGCCGCCGACT-------------------------------------------------------------------TCATGG
TaZIP1-3DL   927 --------------CGTCGCCATCAACCAC-CTCATGGCCAAGGGGT----------------------------------------------------------------------ACC
TaZIP2-6DS   954 --------------CGTCTCCGTCAACCAC-CTCCTCTCCAAGGGGT-------------------------------------------------------------------AC---A
TaZIP3-2DL   957 --------------CATGTCCCTGGTGGAC-CTTCTAGCAACAGATT-------------------------------------------------------------------TCAATA
TaZIP5-4DL   915 --------------CATGGCGCTCGTCGAC-CTTCTAGCGGAAGATT-------------------------------------------------------------------TCATGA
TaZIP6-1DS  1068 --------------CATGGCGCTTGTCGAC-CTCATTTCTCTTGATT-------------------------------------------------------------------TCTTCC
TaZIP7-1DS  1026 --------------CATGGCGCTGGTGGAT-CTAATCGCAGCCGATT-------------------------------------------------------------------TCTTGG
TaZIP8-1DL   951 --------------CATGGCCCTCGTCGAC-CTCCTCGCCGAAGACT-------------------------------------------------------------------TTATGA
TaZIP9-2DS  1002 --------------CATGGCCCTCGTCGAC-CTCCTCGCCGAGGACT-------------------------------------------------------------------TCATGA
TaZIP10-7DL 1098 --------------TATGGCATTAGTGGAT-CTCATTGCTGCTGATT-------------------------------------------------------------------TCCTTA
TaZIP11-1DS 1474 CTTGCGGGACCAAGCGCGGCCATCGGTGCA-ATCCTTG-CGAAGATTGGCTATGATGGACTGGACTATTGGATGGTGATTGCATGCGGGGCTTTGATCCCGAGCTTTGGCCGTGTCTTCA
TaZIP13-2DL  996 --------------CATGGCCCTCGTCGAC-CTCCTCGCCGCGGACT-------------------------------------------------------------------TCATGA
TaZIP14-3DS 1359 --------------CATTGCTGTTGCCGGA-GTCCTC-CCACAGAT--------------------------------------------------------------------GCATGA
TaZIP16-6DS  692 --------------CTTTGCATGAGTTGTTACCTCTAGCATTTGACT-------------------------------------------------------------------ATGCTG

TaIRT1-4DS  1025 GGCCCAAGCT--GCAGGGCAGC----GTCAGGCTCCAGCTCATCTGCCTCACCGCCGTC---CTCCTCGGCGCCGGCGGCATGTCCGT-----------------------CATGGCCAA
TaZIP1-3DL   962 GCCCGCAGCA--GCCAAACTACTTCGACAAGCCCATCTTCAAGTTCC----TCGGCGTG---CTTACCGGCATAGCCGTCATGTCTGT-----------------------CGTCATGAT
TaZIP2-6DS   989 AGCCCCAGCG--GCCCGTCGCC----GCCGACACGCCCCTCGGCCGCTGGCTCGCCGTC---GTGCTCGGCGTCGGGGTCATCGCCGT-----------------------CGTCATGAT
TaZIP3-2DL   995 ACCCAAAGCT--ACAGACAAAT----ACAAAGCTTCAGCTGATGACATATCTTGCACTT---TTCCTAGGTGCAGGGATGATGTCCAT-----------------------GCTTGCCAT
TaZIP5-4DL   953 ATCCTAGGGT--GCAGAACAAC----GGAAGGTTGCAGGTCATCATTAACATCTCCTTG---CTGGTTGGGACGGGTCTCATGTCCAT-----------------------GCTTGCTGT
TaZIP6-1DS  1106 ACAACAAGAT---GATGTCATCATCTCTGAAACTGAAGAAGGCCTCTTATATTGCATTG---GTGCTTGGATCTGCTTCTATGTCGAT-----------------------ATTAGCTCT
TaZIP7-1DS  1064 GTGGGAAGAT------GACAGGGTCGCCACGGCAGCAAGTGATGGCCTATGTCGCCCTG---TTCCTAGGTGCGCTCTCAATGTCATC-----------------------GCTTGCAGT
TaZIP8-1DL   989 ACCCTAGGGT--GCAGGGCAAC----GGAAGGCTGCAGGTCATCGTAAACCTCTCCTTG---CTGCTTGGGACGGGTCTCATGTCAAT-----------------------GCTTGCCAT
TaZIP9-2DS  1040 ACCCTAAGGT--GCAGAGCAGG----GGAAAGCTGCAGCTTGGTATCAACGTCTCCATG---CTGGTCGGTGCAGGCCTCATGTCCAT-----------------------GCTCGCCAA
TaZIP10-7DL 1136 GCCGGAGGAT--GAGCTGCAAT----CCGAGGCTGCAAGTGTGCTCATACGTTGCCTTG---TTTGTCGGGGCTATTGCCATGTCATC-----------------------ACTTGCTAT
TaZIP11-1DS 1592 GACGCTCGCT--GCGGTTGGACGCGAGGAAGAGCGTCTGCGGGCTCCTGATAGGTTTTGGCTTTGCTTGG-GTGTGCTTGATGTCCACCAGATTCATCTGCTTGCACACC-CCTTACTGC
TaZIP13-2DL 1034 ACCCCAAGGT--GCAGAGCAGG----GGGAAGCTGCAGCTTGGCATCAACGTCTCCATG---CTGGTTGGTGCAGGCCTCATGTCCAT-----------------------GCTCGCCAA
TaZIP14-3DS 1395 TCAGAAAACTACGCTAAGCAAC----TCAATGTGTCAGTTGATT------------------TTCCTGACAATGGGAATGCTGGTCGC-----------------------TCTTGGCAT
TaZIP16-6DS  731 GCCAGAAGCA--AGCTGTCAAA-------------------------------GCAGTC---TTTGTTGGCATGGCCGTCATGTCTGCGAGCTTGTATTTCCTGGAGATCAGCCTACCGA

TaIRT1-4DS  1113 A--TGGGC---------------GTGA
TaZIP1-3DL  1050 A--TGGGA---------------CTGA
TaZIP2-6DS  1077 A--TGGGA------------CACCTGA
TaZIP3-2DL  1083 A--TGGGC---------------ATAG
TaZIP5-4DL  1041 A--TGGGC---------------ATGA
TaZIP6-1DS  1197 C--TGGGC---------------ATAG
TaZIP7-1DS  1152 T--TGGGC---------------CTGA
TaZIP8-1DL  1077 A--TGGGC---------------CTGA
TaZIP9-2DS  1128 A--TGGGC---------------CTAG
TaZIP10-7DL 1224 C--TGGGC---------------TTAG
TaZIP11-1DS 1708 AATTCAGGTCCAGAAGCGGTTACATGA
TaZIP13-2DL 1122 A--TGGGC---------------CTAG
TaZIP14-3DS 1470 ATCTTTG----------------GTAG
TaZIP16-6DS  815 A--GGAGATC----AGCTT----GTAA
```
