# Supplementary material for: The role of ZIP transporters and group F bZIP transcription factors in the Zn‐deficiency response of wheat (Triticum aestivum)
Source: Plant J. 2017 Sep 17;92(2):291–304. doi: 10.1111/tpj.13655 (PMC5656842; doi:10.1111/tpj.13655)
Supplement: Supplementary file 3 — Figure S3. Phenotypic effect of Zn starvation throughout a 3‐week growth period. [file TPJ-92-291-s003.pdf]

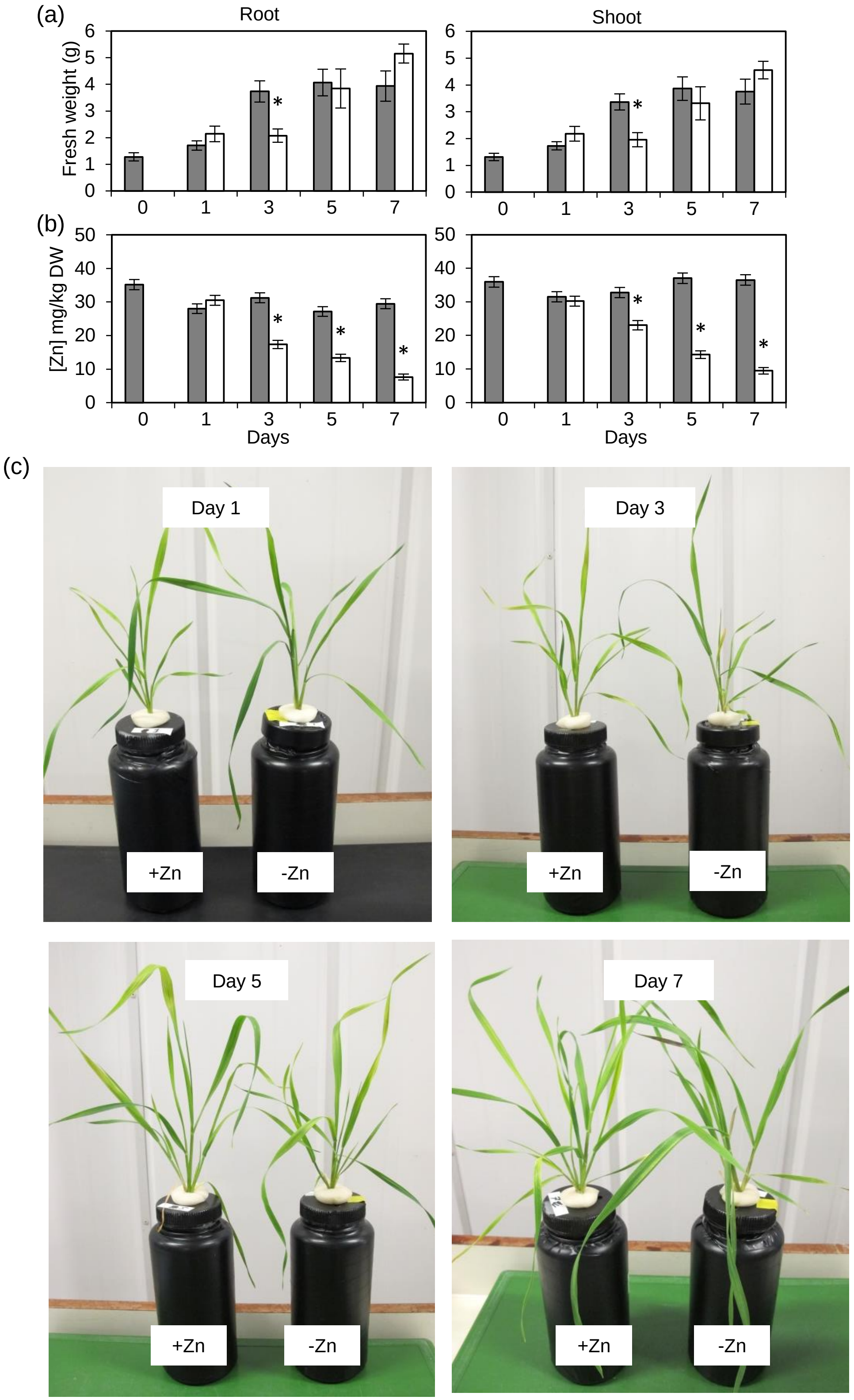

Figure S3. Phenotypic effect of Zn starvation throughout a three-week growth period. The effect of Zn starvation on fresh weight (a) and Zn concentration (b) of wheat roots and shoots. Results shown are means ± S.E.M. (n=6). *<0.05, tested using Fisher's LSD on log-transformed data. +Zn = 8 μM Zn (grey bars), – Zn = 0 μM Zn (white bars). Plants were grown in +Zn culture solution for two weeks before treatment initiation, then sampled at days 0, 1, 3, 5 and 7 following –Zn treatment initiation. (c) No visible phenotypes were observed in shoot growth throughout the one-week starvation period. +Zn=8 μM, -Zn=0 μM.
