# Supplementary material for: The role of ZIP transporters and group F bZIP transcription factors in the Zn‐deficiency response of wheat (Triticum aestivum)
Source: Plant J. 2017 Sep 17;92(2):291–304. doi: 10.1111/tpj.13655 (PMC5656842; doi:10.1111/tpj.13655)
Supplement: Supplementary file 4 — Figure S4. Mineral concentration analysis of wheat root and shoot samples during a 1‐week Zn‐ starvation period. [file TPJ-92-291-s004.pdf]

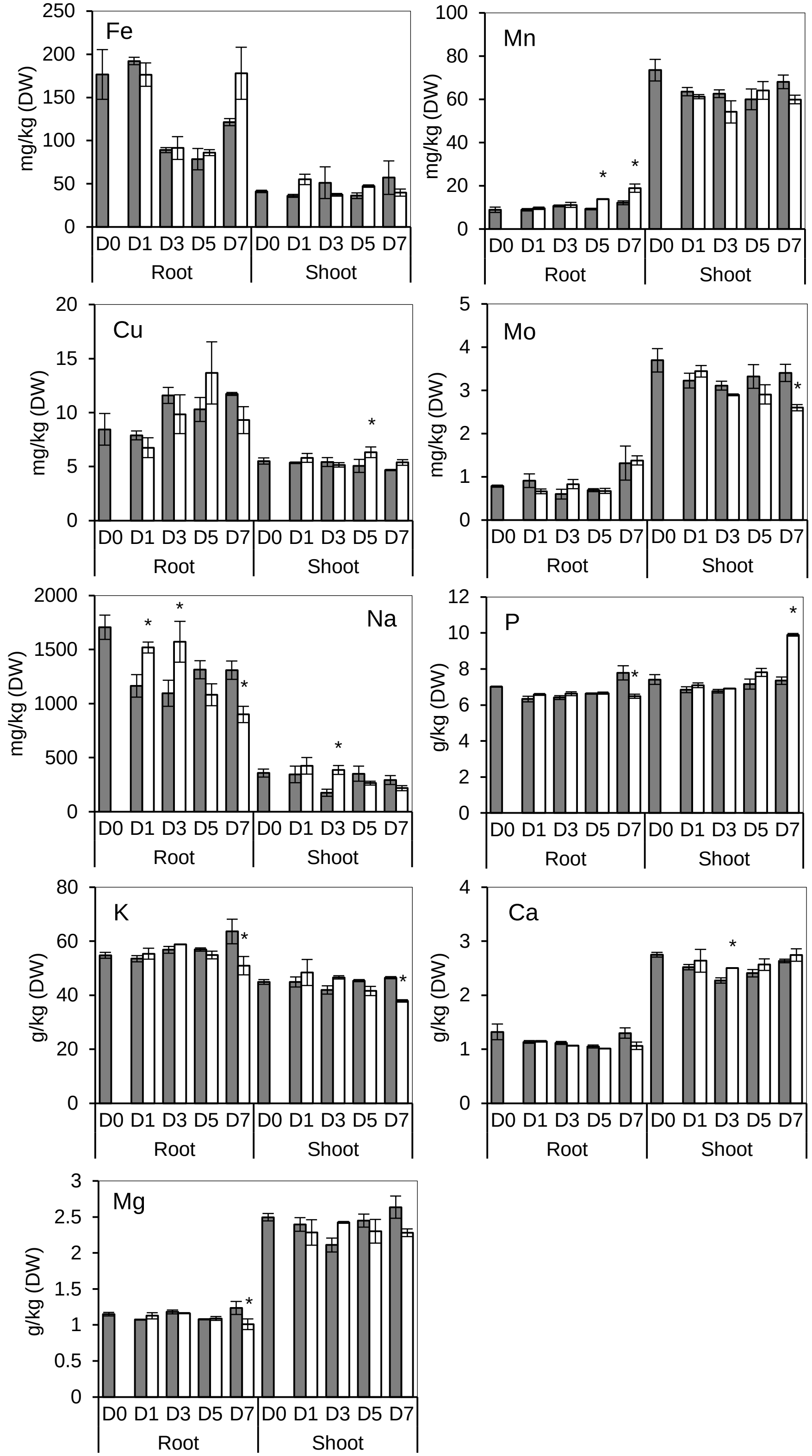

Figure S4. Mineral concentration analysis of wheat root and shoot samples during a one-week Zn- starvation period. Results shown are means ± S.E.M. (n=3). *<0.05, tested using Fisher's LSD on log-transformed data. +Zn = 8 µM Zn (grey bars), – Zn = 0 µM Zn (white bars).
