# Supplementary material for: The role of ZIP transporters and group F bZIP transcription factors in the Zn‐deficiency response of wheat (Triticum aestivum)
Source: Plant J. 2017 Sep 17;92(2):291–304. doi: 10.1111/tpj.13655 (PMC5656842; doi:10.1111/tpj.13655)
Supplement: Supplementary file 5 — Figure S5. Gene expression analysis of TaZIPs in wheat root and shoot material throughout an extended Zn starvation period of 3 weeks. [file TPJ-92-291-s005.pdf]

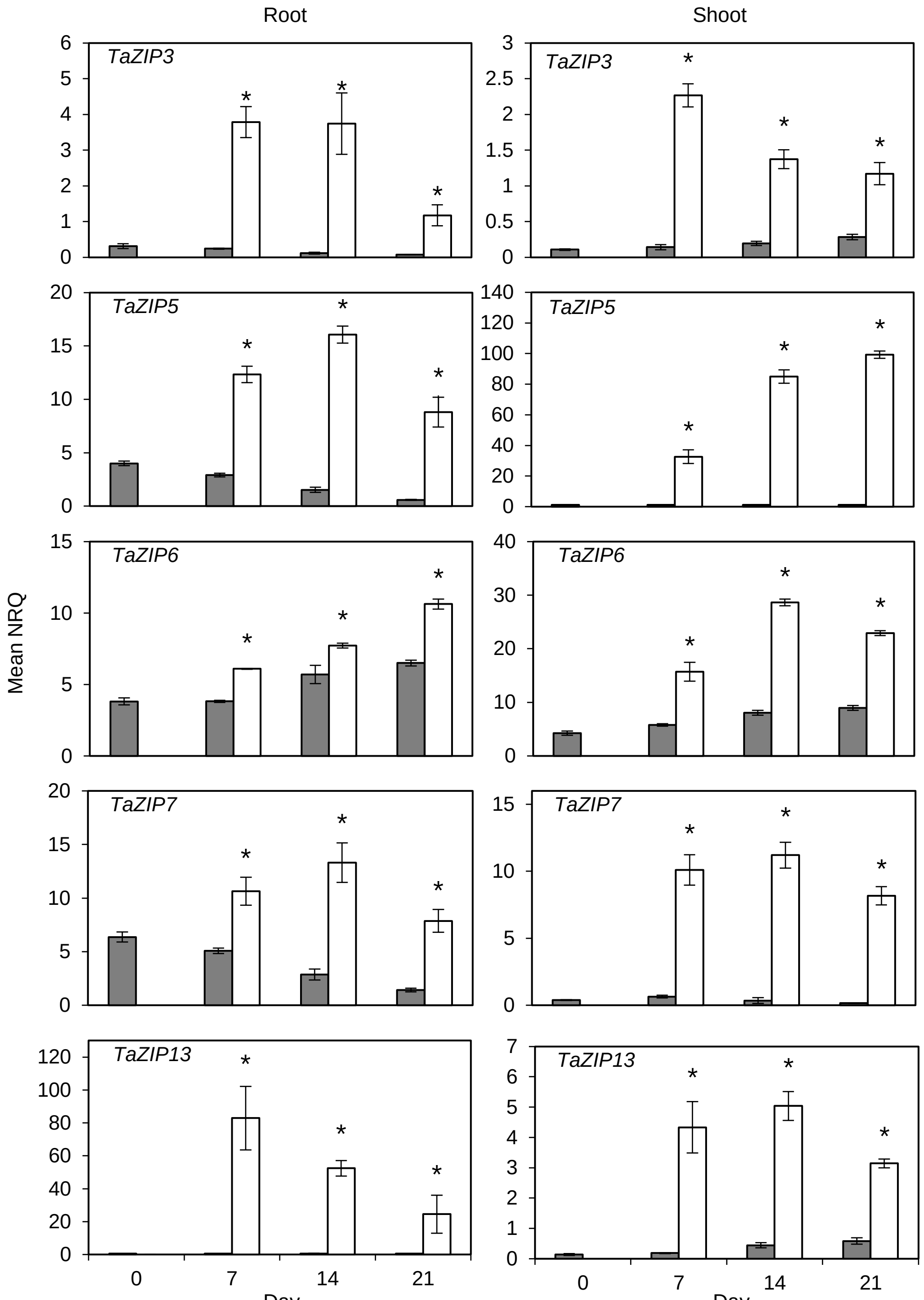

Figure S5. Gene expression analysis of *TaZIPs* in wheat root and shoot material throughout an extended Zn starvation period of three weeks. Relative expression levels (Normalized relative quantification (NRQ)) of five wheat *ZIP* transporter gene transcripts in root and shoot material throughout one week of Zn starvation. NRQ values are normalised to *TaActin3* expression, means of three biological replicates are given (± S.E.M). Bars within individual graphs displaying an asterisk show significant difference between treatment means at a given time point. Significance ($P<0.05$) was tested post-hoc, using Fisher's LSD test on Log2(1/NRQ) transformed data. +Zn = 8 µM Zn (grey bars), – Zn = 0 µM Zn (white bars).
