# Supplementary material for: The role of ZIP transporters and group F bZIP transcription factors in the Zn‐deficiency response of wheat (Triticum aestivum)
Source: Plant J. 2017 Sep 17;92(2):291–304. doi: 10.1111/tpj.13655 (PMC5656842; doi:10.1111/tpj.13655)
Supplement: Supplementary file 7 — Figure S7. Confirmatory PCR of 35S::TabZIP‐transformed Arabidopsis lines. [file TPJ-92-291-s007.pdf]

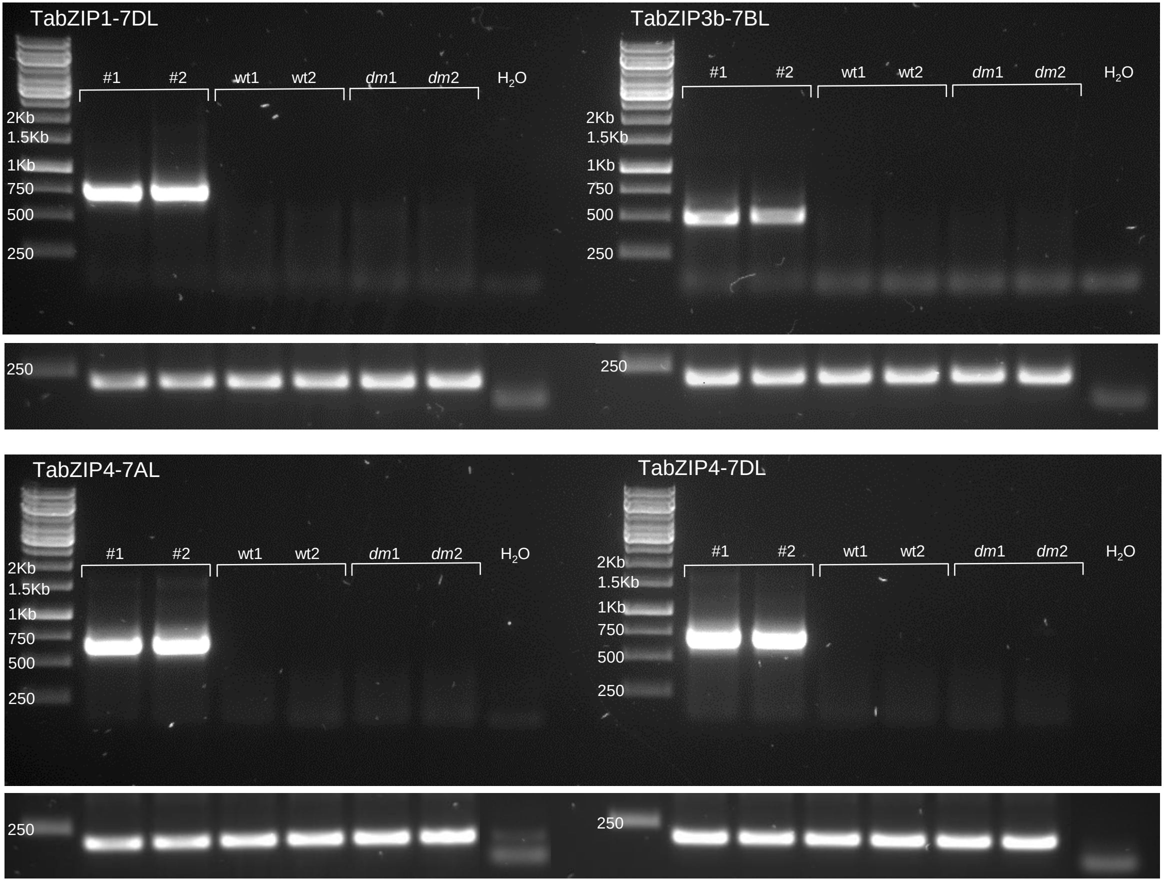

Figure S7. Confirmatory PCR of 35S::2TabZIP transformed *Arabidopsis* lines. Confirmatory PCR reactions showing the presence of each of the four *TabZIPs* in the transformed *Arabidopsis bzip19 bzip23* line. For each TabZIP, cDNA from two individual lines were tested (#1 and #2). Amplifications were carried out with corresponding full length amplification primers detailed in Table S4 primers, expected sizes are as follows: TabZIP1-7DL=757 bp, TabZIP3b-7BL=531 bp, TabZIP4-7AL=712 bp and TabZIP4-7DL=712 bp. Two individual wild type cDNA preparations (wt1 and wt2), two individual double mutant *bzip19-4 bzip23-2* cDNA preparations (dm1 and dm2) as well as a blank water control (H_2O) were also tested with the corresponding TabZIPTOPO primers as control reactions. All cDNA preparations were also tested with AtActin2 primers, these amplifications are shown below the main gels and correspond to samples in the gels as labelled directly above. Expected size of AtActin2 primer amplicon is 201 bp. DNA ladder used in all gels is Thermo Scientific GeneRuler 1 kb.
