# Supplementary material for: The role of ZIP transporters and group F bZIP transcription factors in the Zn‐deficiency response of wheat (Triticum aestivum)
Source: Plant J. 2017 Sep 17;92(2):291–304. doi: 10.1111/tpj.13655 (PMC5656842; doi:10.1111/tpj.13655)
Supplement: Supplementary file 8 — Table S1. TaZIP identity matrix. [file TPJ-92-291-s008.docx]

Table S1. TaZIP identity matrix. The percentage of bases/residues which are identical in the coding sequence are given, those shown in bold refer to the nucleotide sequences, those in italics refer to amino acid translations. Identities are shaded according to sequence similarity; darker grey indicates higher similarity.

|  | TaIRT1-4DS | | TaZIP1-3DL | TaZIP2-6DS | TaZIP3-2DL | TaZIP5-4DL | TaZIP6-1DS | TaZIP7-1DS | TaZIP8-1DL | TaZIP9-2DS | TaZIP10-7DL | TaZIP11-1DS | TaZIP13-2DL | TaZIP14-3DS | TaZIP16-6DS |
| --- | --- | --- | --- | --- | --- | --- | --- | --- | --- | --- | --- | --- | --- | --- | --- |
| TaIRT1-4DS | |  | **36.44** | **42.38** | **48.90** | **54.54** | **43.12** | **50.25** | **57.87** | **54.29** | **46.96** | **24.55** | **54.83** | **30.01** | **27.07** |
| TaZIP1-3DL | | *16.93* |  | **49.80** | **35.20** | **38.19** | **30.01** | **34.08** | **38.96** | **38.80** | **31.17** | **19.75** | **36.77** | **25.50** | **25.09** |
| TaZIP2-6DS | | *18.44* | *48.39* |  | **37.53** | **40.27** | **33.33** | **36.13** | **43.53** | **39.70** | **34.84** | **21.78** | **38.46** | **25.05** | **25.04** |
| TaZIP3-2DL | | *41.89* | *18.24* | *21.01* |  | **55.62** | **40.60** | **49.09** | **55.79** | **55.98** | **45.08** | **25.25** | **55.20** | **30.38** | **27.75** |
| TaZIP5-4DL | | *43.83* | *17.53* | *18.43* | *53.16* |  | **42.96** | **49.17** | **80.84** | **59.23** | **47.41** | **24.20** | **58.58** | **29.32** | **31.37** |
| TaZIP6-1DS | | *30.67* | *13.62* | *16.08* | *31.71* | *30.50* |  | **44.98** | **44.95** | **47.21** | **45.78** | **24.55** | **46.36** | **27.25** | **26.42** |
| TaZIP7-1DS | | *41.86* | *17.04* | *17.01* | *42.99* | *41.12* | *34.32* |  | **49.96** | **50.73** | **52.87** | **25.62** | **49.88** | **29.95** | **27.78** |
| TaZIP8-1DL | | *44.84* | *17.98* | *18.93* | *52.30* | *79.04* | *31.81* | *41.62* |  | **61.43** | **49.22** | **25.01** | **60.10** | **29.30** | **29.88** |
| TaZIP9-2DS | | *41.58* | *16.79* | *17.17* | *50.51* | *53.78* | *35.11* | *41.39* | *53.95* |  | **49.49** | **24.20** | **83.35** | **29.46** | **28.27** |
| TaZIP10-7DL | | *33.53* | *15.57* | *17.10* | *37.89* | *37.26* | *32.98* | *57.02* | *38.92* | *40.74* |  | **24.70** | **49.34** | **29.00** | **25.21** |
| TaZIP11-1DS | | *10.75* | *9.97* | *10.31* | *9.81* | *10.36* | *10.82* | *11.41* | *11.34* | *11.34* | *10.15* |  | **23.47** | **23.27** | **18.38** |
| TaZIP13-2DL | | *42.73* | *17.21* | *17.39* | *52.35* | *53.68* | *33.85* | *42.95* | *54.28* | *82.82* | *41.81* | *11.19* |  | **29.44** | **29.12** |
| TaZIP14-3DS | | *9.56* | *10.34* | *11.13* | *12.72* | *12.75* | *11.13* | *11.16* | *12.55* | *12.75* | *10.20* | *8.91* | *12.30* |  | **18.39** |
| TaZIP16-6DS | | *12.93* | *13.08* | *12.88* | *14.38* | *16.05* | *13.31* | *14.16* | *14.89* | *13.06* | *12.65* | *10.69* | *13.10* | *11.00* |  |
