# Supplementary material for: The role of ZIP transporters and group F bZIP transcription factors in the Zn‐deficiency response of wheat (Triticum aestivum)
Source: Plant J. 2017 Sep 17;92(2):291–304. doi: 10.1111/tpj.13655 (PMC5656842; doi:10.1111/tpj.13655)
Supplement: Supplementary file 9 — Table S2. Wheat TaZIP and TabZIP gene identification details. [file TPJ-92-291-s009.docx]

Table S2. Wheat TaZIP and TabZIP gene identification details. Gene name indicates nomenclature used in this study, with sequence identifiers for both the Plant Ensemble ID and the TGAC scaffolds given. (-) indicates a homeolog or gene not previously described. (N.F.) indicates a likely non-functional translation caused by a frameshift in the coding sequence.

| Gene name | Plant Ensemble ID | Previously published gene name | Reference |
| --- | --- | --- | --- |
| *TaIRT1-4AL* | TRIAE_CS42_4AL_TGACv1_290140_AA0982100 | - |  |
| *TaIRT1-4BS* | TRIAE_CS42_4BS_TGACv1_328611_AA1090980 | - |  |
| *TaIRT1-4DS* | TRIAE_CS42_4DS_TGACv1_361462_AA1168460 | TaIRT1 | Tiong et al*.,* 2015 |
| *TaZIP1-3AL* | TRIAE_CS42_3AL_TGACv1_195235_AA0646850 | - |  |
| *TaZIP1-3BL* | TRIAE_CS42_3B_TGACv1_221732_AA0748740 | TaZIP1 | Tiong et al*.,* 2015 |
| *TaZIP1-3DL* | TRIAE_CS42_3DL_TGACv1_250330_AA0866240 | - |  |
| *TaZIP2-6AS* | TRIAE_CS42_6AS_TGACv1_487184_AA1568790 | - |  |
| *TaZIP2-6BS* | TRIAE_CS42_6BS_TGACv1_513631_AA1646150 | - |  |
| *TaZIP2-6DS* | TRIAE_CS42_6DS_TGACv1_544682_AA1749140 | - |  |
| *TaZIP3-2AL* | TRIAE_CS42_2AL_TGACv1_094530_AA0299310 | TaZIP3 | Tiong et al, 2015 |
| *TaZIP3-2BL* | TRIAE_CS42_2BL_TGACv1_129658_AA0391920 | - |  |
| *TaZIP3-2DL* | TRIAE_CS42_2DL_TGACv1_160080_AA0546500 | - |  |
| *TaZIP5-4AS* | TRIAE_CS42_4AS_TGACv1_306183_AA1003790 | TaZIP5 | Tiong et al*.,* 2015 |
| *TaZIP5-4BL* | TRIAE_CS42_4BL_TGACv1_321083_AA1054990 | - |  |
| *TaZIP5-4DL* | TRIAE_CS42_4DL_TGACv1_342890_AA1124750 | - |  |
| *TaZIP6-1AS* | TRIAE_CS42_1AS_TGACv1_019973_AA0073380 | - |  |
| *TaZIP6-1BS* | TRIAE_CS42_1BS_TGACv1_050073_AA0167090 | TaZIP6 | Tiong et al*.,* 2015 |
| *TaZIP6-1DS* | TRIAE_CS42_1DS_TGACv1_082083_AA0263760 | - |  |
| *TaZIP7-1AS* (N.F.) | TRIAE_CS42_1AS_TGACv1_018996_AA0057560 | - |  |
| *TaZIP7-1BS* | TRIAE_CS42_1BS_TGACv1_049369_AA0150920 | - |  |
| *TaZIP7-1DS* | TRIAE_CS42_1DS_TGACv1_082535_AA0264750 | TaZIP7 | Tiong et al*.,* 2015 |
| *TaZIP8-1AL* | TRIAE_CS42_1AL_TGACv1_000915_AA0021780 | - |  |
| *TaZIP8-1BL* | TRIAE_CS42_1BL_TGACv1_030324_AA0086740 | - |  |
| *TaZIP8-1DL* | TRIAE_CS42_1DL_TGACv1_061188_AA0188250 | - |  |
| *TaZIP9-2AS* | TRIAE_CS42_2AS_TGACv1_112113_AA0330750 | - |  |
| *TaZIP9-2BS* | TRIAE_CS42_2BS_TGACv1_146518_AA0467340 | - |  |
| *TaZIP9-2DS* | TRIAE_CS42_2DS_TGACv1_177487_AA0578500 | - |  |
| *TaZIP10-7AL* | TRIAE_CS42_7AL_TGACv1_558847_AA1796430 | TaZIP10 | Tiong et al*.,* 2015 |
| *TaZIP10-7BL* | TRIAE_CS42_7BL_TGACv1_577920_AA1886220 | - |  |
| *TaZIP10-7DL* | TRIAE_CS42_7DL_TGACv1_603327_AA1981040 | - |  |
| *TaZIP11-1AS* | TRIAE_CS42_1AS_TGACv1_019215_AA0063260 | TaZIP11 | Tiong et al*.,* 2015 |
| *TaZIP11-1BS* | TRIAE_CS42_1BS_TGACv1_049922_AA0164330 | - |  |
| *TaZIP11-1DS* | TRIAE_CS42_1DS_TGACv1_080155_AA0241650 | - |  |
| *TaZIP13-2AL* | TRIAE_CS42_2AL_TGACv1_098072_AA0325680 | - |  |
| *TaZIP13-2BL* | TRIAE_CS42_2BL_TGACv1_129838_AA0397670 | - |  |
| *TaZIP13-2DL* | TRIAE_CS42_2DL_TGACv1_159221_AA0534750 | TaZIP13 | Tiong et al*.,* 2015 |
| *TaZIP14-3AS* | TRIAE_CS42_3AS_TGACv1_212290_AA0699680 | - |  |
| *TaZIP14-3BS* | TRIAE_CS42_3B_TGACv1_224841_AA0802240 | - |  |
| *TaZIP14-3DS* | TRIAE_CS42_3DS_TGACv1_273454_AA0931350 | TaZIP14 | Tiong et al*.,* 2015 |
| *TaZIP16-6AS* | TRIAE_CS42_6AS_TGACv1_485332_AA1543320 | TaZIP16 | Tiong et al*.,* 2015 |
| *TaZIP16-6BS* | TRIAE_CS42_6BS_TGACv1_513963_AA1653060 | - |  |
| *TaZIP16-6DS* | TRIAE_CS42_6DS_TGACv1_542944_AA1733060 | - |  |
| *TabZIPF1-7AL* | TRIAE_CS42_U_TGACv1_641258_AA2089960 | TabZIP56 | Li et al*.,* 2015 |
| *TabZIPF1-7BL* | TRIAE_CS42_7BL_TGACv1_577539_AA1878150 | - |  |
| *TabZIPF1-7DL* | TRIAE_CS42_7DL_TGACv1_604895_AA2002830 | TabZIP180 | Li et al*.,* 2015 |
| *TabZIPF2-5AS* | TRIAE_CS42_5AS_TGACv1_393100_AA1268400 | TabZIP33 | Li et al*.,* 2015 |
| *TabZIPF2-5BS* | TRIAE_CS42_5BS_TGACv1_423566_AA1379610 | TabZIP97 | Li et al*.,* 2015 |
| *TabZIPF2-5DS* | TRIAE_CS42_5DS_TGACv1_456500_AA1472280 | TabZIP150 | Li et al*.,* 2015 |
| *TabZIPF3a-7AL* | TRIAE_CS42_7AL_TGACv1_557389_AA1780670 | - |  |
| *TabZIPF3a-7BL* | TRIAE_CS42_7BL_TGACv1_577387_AA1874120 | - |  |
| *TabZIPF3a-7DL* | TRIAE_CS42_7DL_TGACv1_602722_AA1966420 | TabZIP179 | Li et al*.,* 2015 |
| *TabZIPF3b-7AL* | TRIAE_CS42_7AL_TGACv1_556472_AA1763720 | - |  |
| *TabZIPF3b-7BL* | TRIAE_CS42_7BL_TGACv1_577539_AA1878160 | - |  |
| *TabZIPF3b-7DL* | - | - |  |
| *TabZIPF4-7AL* | TRIAE_CS42_7AL_TGACv1_558616_AA1794710 | TabZIP55 | Li et al*.,* 2015 |
| *TabZIPF4-7BL* | TRIAE_CS42_7BL_TGACv1_577103_AA1866000 | - |  |
| *TabZIPF4-7DL* | TRIAE_CS42_7DL_TGACv1_603100_AA1975830 | - |  |
| *TabZIPF5-1AL* | TRIAE_CS42_1AL_TGACv1_001373_AA0029470 | TabZIP4 | Li et al*.,* 2015 |
| *TabZIPF5-1BL* | TRIAE_CS42_1BL_TGACv1_032488_AA0130420 | TabZIP62 | Li et al*.,* 2015 |
| *TabZIPF5-1DL* | - | - |  |
| *TabZIPF6-3AL*(N.F.) | TRIAE_CS42_3AL_TGACv1_196209_AA0658260 | - |  |
| *TabZIPF6-3BL* | TRIAE_CS42_3B_TGACv1_221777_AA0749730 | TabZIP79 | Li et al*.,* 2015 |
| *TabZIPF6-3DL* (N.F.) | TRIAE_CS42_3DL_TGACv1_251113_AA0877600 | - |  |
