# Supplementary material for: The role of ZIP transporters and group F bZIP transcription factors in the Zn‐deficiency response of wheat (Triticum aestivum)
Source: Plant J. 2017 Sep 17;92(2):291–304. doi: 10.1111/tpj.13655 (PMC5656842; doi:10.1111/tpj.13655)
Supplement: Supplementary file 10 — Table S3. Overview of ZDREs present in promoters of TaZIPs. [file TPJ-92-291-s010.docx]

Table S3. Overview of ZDREs present in promoters of *TaZIPs*. The number, sequence and position of ZDREs with up to one mismatch (shown in red) from the consensus described by (Assunção et al., 2010) of RTGTCGACAY are shown. N.F. indicates a likely non-functional translation caused by a frameshift in the coding sequence. 2000bp of promoters were used in this ZDRE analysis unless stated otherwise.

| **Gene name** | **No. of ZDRE** | **Putative ZDRE sequences** | **ZDRE position from start codon** |
| --- | --- | --- | --- |
| *AtZIP4* | 2 | ATGTCGACAC,ATGTCGACAT | 118bp, 246bp |
| *TaIRT1-4AL* | 0 | - | - |
| *TaIRT1-4BS* | 0 | - | - |
| *TaIRT1-4DS* | 0 | - | - |
| *TaZIP1-3AL* | 1 | GTCTCGACAT | 488bp |
| *TaZIP1-3BL* | 1 | GTCTCGACAT | 621bp |
| *TaZIP1-3DL* | 2 | ATCTCGACAT,ATGTCAACAC | 921bp, 1590bp |
| *TaZIP2-6AS* | 0 | - | - |
| *TaZIP2-6BS* | 0 | - | - |
| *TaZIP2-6DS* | 0 | - | - |
| *TaZIP3-2AL* | 2 | GTGTCAACAC,ATGACGACAT | 489bp, 500bp |
| *TaZIP3-2BL* | 2 | GTGTCAACAC,ATGACGACAT | 570bp, 581bp |
| *TaZIP3-2DL* | 2 | GTGTCAACAC,ATGACGACAT | 554bp, 565bp |
| *TaZIP5-4AS* | 3 | ATGTTGACAT,GTGTCGACAC,GTGTCGACAC | 123bp, 765bp, 985bp |
| *TaZIP5-4BL* | 3 | ATGTTGACAT,GTGTCGACAC,GTGTCGACAC | 121bp, 1076bp, 1294bp |
| *TaZIP5-4DL* | 3 | ATGTTGACAT,GTGTCGACAC,GTGTCGACAC | 121bp, 830bp, 1051bp |
| *TaZIP6-1AS* | 1 | ATGTCGAGAC | 1107bp |
| *TaZIP6-1BS* | 0 | - | - |
| *TaZIP6-1DS* | 0 | - | - |
| *TaZIP7-1AS* (N.F.) | 2 | ATGACGACAC,ATGTCGACAT | 163bp, 640bp |
| *TaZIP7-1BS* | 2 | ATGACGACAC,ATGTCGACAT | 163bp, 644bp |
| *TaZIP7-1DS* | 2 | ATGACGACAC,ATGTCGACAT | 163bp, 625bp |
| *TaZIP8-1AL* | 1 | GTGTCGACAC | 689bp |
| *TaZIP8-1BL* | 1 | GTGTCGACAC | 646bp |
| *TaZIP8-1DL* | 1 | GTGTCGACAC | 663bp, (1291bp of promoter available) |
| *TaZIP9-2AS* | 1 | GTGTCGTCAT | 378bp |
| *TaZIP9-2BS* | 2 | ATGTCGTCAC,GTGTCGTCAT | 241bp, 376bp |
| *TaZIP9-2DS* | 2 | ATGTCGTCAC,GTGTCGTCAT | 234bp, 370bp |
| *TaZIP10-7AL* | 2 | ATGACGACAC,GTGTCGACAT | 280bp, 528bp |
| *TaZIP10-7BL* | 2 | ATGACGACAC,GTGTCGACAT | 281bp, 527bp |
| *TaZIP10-7DL* | 2 | ATGACGACAC,GTGTCGACAT | 393bp, 639bp |
| *TaZIP11-1AS* | 1 | ATGTCGACTT | 175bp |
| *TaZIP11-1BS* | 0 | - | - |
| *TaZIP11-1DS* | 1 | ATGTCGACTT | 175bp |
| *TaZIP13-2AL* | 2 | ATGTCGTCAC,GTGTCGTCAT | 229bp, 373bp |
| *TaZIP13-2BL* | 2 | ATGTCGTCAC,GTGTCGTCAT | 231bp, 374bp |
| *TaZIP13-2DL* | 1 | ATGTCGTCAC | 245bp |
| *TaZIP14-3AS* | 0 | - | - |
| *TaZIP14-3BS* | 0 | - | - |
| *TaZIP14-3DS* | 0 | - | - |
| *TaZIP16-6AS* | 0 | - | - |
| *TaZIP16-6BS* | 0 | - | - |
| *TaZIP16-6DS* | 0 | - | - |
