# Supplementary material for: The role of ZIP transporters and group F bZIP transcription factors in the Zn‐deficiency response of wheat (Triticum aestivum)
Source: Plant J. 2017 Sep 17;92(2):291–304. doi: 10.1111/tpj.13655 (PMC5656842; doi:10.1111/tpj.13655)
Supplement: Supplementary file 11 — Table S4. Oligonucleotide primer sequences used for cloning of full length TaZIPs and TabZIPs. [file TPJ-92-291-s011.docx]

Table S4. Oligonucleotide primer sequences used for cloning of full length *TaZIPs* and *TabZIPs.*

| Gene | Forward primer | Reverse primer |
| --- | --- | --- |
| *TaZIP3* | CACCATGGGCGCCACCAATC | CTATGCCCATATGGCAAGCATGGA |
| *TaZIP6* | CACCATGTCCGGCAAGGGTTG | CTATGCCCAGAGAGCTAATATCGACA |
| *TaZIP7* | CACCATGATGATCGGTGTCGC | TCAGGCCCAAACTGCAAGCG |
| *TaZIP9* | CACCATGAAGCCGAGCGCCG | CTAGGCCCATTTGGCGAGCA |
| *TaZIP13* | CACCATGAAGCCGAGCGCCG | CTAGGCCCATTTGGCGAGCA |
| *TabZIPF1* | CACCATGGACGACGGGGACATC | TTACAGCATTTGGCCCCCACG |
| *TabZIPF3b* | CACCATGGACGACGGGAACCTC | TTAAAGAAAACACGTATGAGGTTGTT |
| *TabZIPF4* | CACCATGGACGACGGGGACATA | TCACCTCTTTACATCATCTGGCAAA |
