# Supplementary material for: The role of ZIP transporters and group F bZIP transcription factors in the Zn‐deficiency response of wheat (Triticum aestivum)
Source: Plant J. 2017 Sep 17;92(2):291–304. doi: 10.1111/tpj.13655 (PMC5656842; doi:10.1111/tpj.13655)
Supplement: Supplementary file 12 — Table S5. Oligonucleotide primer sequences used for SYBR Green real time RT‐PCR expression analysis. [file TPJ-92-291-s012.docx]

Table S5. Oligonucleotide primer sequences used for SYBR Green real time RT-PCR expression analysis.

| Gene symbol | Forward primer | Reverse primer |
| --- | --- | --- |
| *TaZIP3* | GGGAAATGGAGAACYCCTGGATG | GGCATAGAGATCTTGAAAGCAATTGC |
| *TaZIP5* | AGGTTTCGCCTCAAGTCTGTCTTG | GGCTATTCTCGTCGTAAGCAGAG |
| *TaZIP6* | GTCATCATCTCTGAAACTGAAGAAGG | CCCTCTATACATTTCACTATGRCC |
| *TaZIP7* | ACAGGCAGTATGTTSGGACGTAG | CAGCAAGTGATGGCCTATGTCG |
| *TaZIP13* | CGCAAGCSTACAACATGAAACAGT and CGCGAGCCTACAACTTGAAACAG | CTTYAGACACGCTACTGGGTTGG |
| *TabZIPF1* | CAAGGTGCYGGTGACTCTATGG | TCCTCGACCTGTTTACAGCATTTG |
| *TabZIPF3a* | TCTSAGAAGGCAACGTATCCARAG | CATGATGAGCTCATGTGGCTTCGT |
| *TabZIPF3b* | TGCCCAGGTTATGAGCTCATGTG | TTCTCCATTGCATAGATCTGCTCTG |
| *TabZIPF4* | CGAGSTGTTGGGTCAAGSTGC | CCTCTTTACATCATCKGGCAAACC |
| *TaActin3* | GACGCACAACAGGTATCGTGTTG | CAGCGAGGTCAAGACGAAGGATG |
| *TaSuccDH* | TTTGCTCTCCGTGGTGCCTTTGG | GAAGATGTGTAGCTCCTTGCTTGC |
