# Supplementary material for: The role of ZIP transporters and group F bZIP transcription factors in the Zn‐deficiency response of wheat (Triticum aestivum)
Source: Plant J. 2017 Sep 17;92(2):291–304. doi: 10.1111/tpj.13655 (PMC5656842; doi:10.1111/tpj.13655)
Supplement: Supplementary file 13 — Table S6. Oligonucleotide primer sequences used for PCR‐amplification of TabZIPs. With SP6 promoters and Poly‐A tails prior to in vitro transcription translation. [file TPJ-92-291-s013.docx]

Table S6. Oligonucleotide primer sequences used for PCR-amplification of TabZIPs. With SP6 promoters and Poly-A tails prior to in vitro transcription translation.

| Gene | Forward primer (with SP6 promoter) | Reverse primer (with poly-A tail) |
| --- | --- | --- |
| TabZIPF1 | GACTCATATTTAGGTGACACTATAGAACAGACCACCATGGACGACGGGGA | TTTTTTTTTTTTTTTTTTTTTTTTTTTTTTTTACAGCATTTGGCCCCC |
| TabZIPF3b | GACTCATATTTAGGTGACACTATAGAACAGACCACCATGGACGACGGGAA | TTTTTTTTTTTTTTTTTTTTTTTTTTTTTAAAGAAAACACGTATGAGGT |
| TabZIPF4 | GACTCATATTTAGGTGACACTATAGAACAGACCACCATGGACGACGGGGA | TTTTTTTTTTTTTTTTTTTTTTTTTTTTTCACCTCTTTACATCATCTGG |
| AtbZIP19 | GACTCATATTTAGGTGACACTATAGAAGACCACCATGGAAGACGGTGAG | TTTTTTTTTTTTTTTTTTTTTTTTTTTTTTTCAAACTGCTCTTGATGCAC |
