# Supplementary material for: The role of ZIP transporters and group F bZIP transcription factors in the Zn‐deficiency response of wheat (Triticum aestivum)
Source: Plant J. 2017 Sep 17;92(2):291–304. doi: 10.1111/tpj.13655 (PMC5656842; doi:10.1111/tpj.13655)
Supplement: Supplementary file 14 — Table S7. Complementary oligonucleotides used in EMSAs. [file TPJ-92-291-s014.docx]

Table S7. Complementary oligonucleotides used in EMSAs.

| Probe | Forward primer | Reverse primer |
| --- | --- | --- |
| Ass3Z | biotinAATTCATGTCGACATATGTCGACATATGTCGACACGAGCT | AGCTCGTGTCGACATATGTCGACATATGTCGACATGAATT |
| Ass3Zmut | biotinAATTCATGTAGACATATGTAGACATATGTAGACACGAGCT | AGCTCGTGTCTACATATGTCTACATATGTCTACATGAATT |
| Ass2Z | biotinAATTCATGTCGACATATGTCGACACGAGCT | AGCTCGTGTCGACATATGTCGACATGAATT |
| TaZIP3ZDRE | biotinGACCGATGACGACATTGTGTCAACACTGCAC | GTGCAGTGTTGACACAATGTCGTCATCGGTC |
| TaZIP4ZDRE | biotinAATTCGTGTCGACACGTGTCGACACATGTTGACATGAGCT | AGCTCATGTCAACATGTGTCGACACGTGTCGACACGAATT |
| TaZIP5ZDRE | biotinAATTCGTGTCGTCATATGTCGTCACGAGCT | AGCTCGTGACGACATATGACGACACGAATT |
| TaZIP6ZDRE | biotinAATTCATGTCGAGACATGTCGAGACGAGCT | AGCTCGTCTCGACATGTCTCGACATGAATT |
| TaZIP7ZDRE | biotinAATTCATGTCGACATATGACGACACGAGCT | AGCTCGTGTCGTCATATGTCGACATGAATT |
