# Supplementary material for: The role of ZIP transporters and group F bZIP transcription factors in the Zn‐deficiency response of wheat (Triticum aestivum)
Source: Plant J. 2017 Sep 17;92(2):291–304. doi: 10.1111/tpj.13655 (PMC5656842; doi:10.1111/tpj.13655)
Supplement: Supplementary file 15 [file TPJ-92-291-s015.docx]

**Table S1.** TaZIP identity matrix.

**Table S2.** TaZIP identity matrix.

**Table S3.** Overview of ZDREs present in promoters of *TaZIPs*.

**Table S4.** Oligonucleotide primer sequences used for cloning of full length *TaZIPs* and *TabZIPs.*

**Table S5.** Oligonucleotide primer sequences used for SYBR Green real time RT-PCR expression analysis.

**Table S6.** Oligonucleotide primer sequences used for PCR-amplification of TabZIPs. With SP6 promoters and Poly-A tails prior to in vitro transcription translation.

**Table S7.** Complementary oligonucleotides used in EMSAs.
