# Supplementary material for: The role of ZIP transporters and group F bZIP transcription factors in the Zn‐deficiency response of wheat (Triticum aestivum)
Source: Plant J. 2017 Sep 17;92(2):291–304. doi: 10.1111/tpj.13655 (PMC5656842; doi:10.1111/tpj.13655)
Supplement: Supplementary file 6 — Figure S6. Multiple sequence alignment of cloned group F TabZIPs, AtbZIP19 and AtbZIP23. [file TPJ-92-291-s006.pdf]

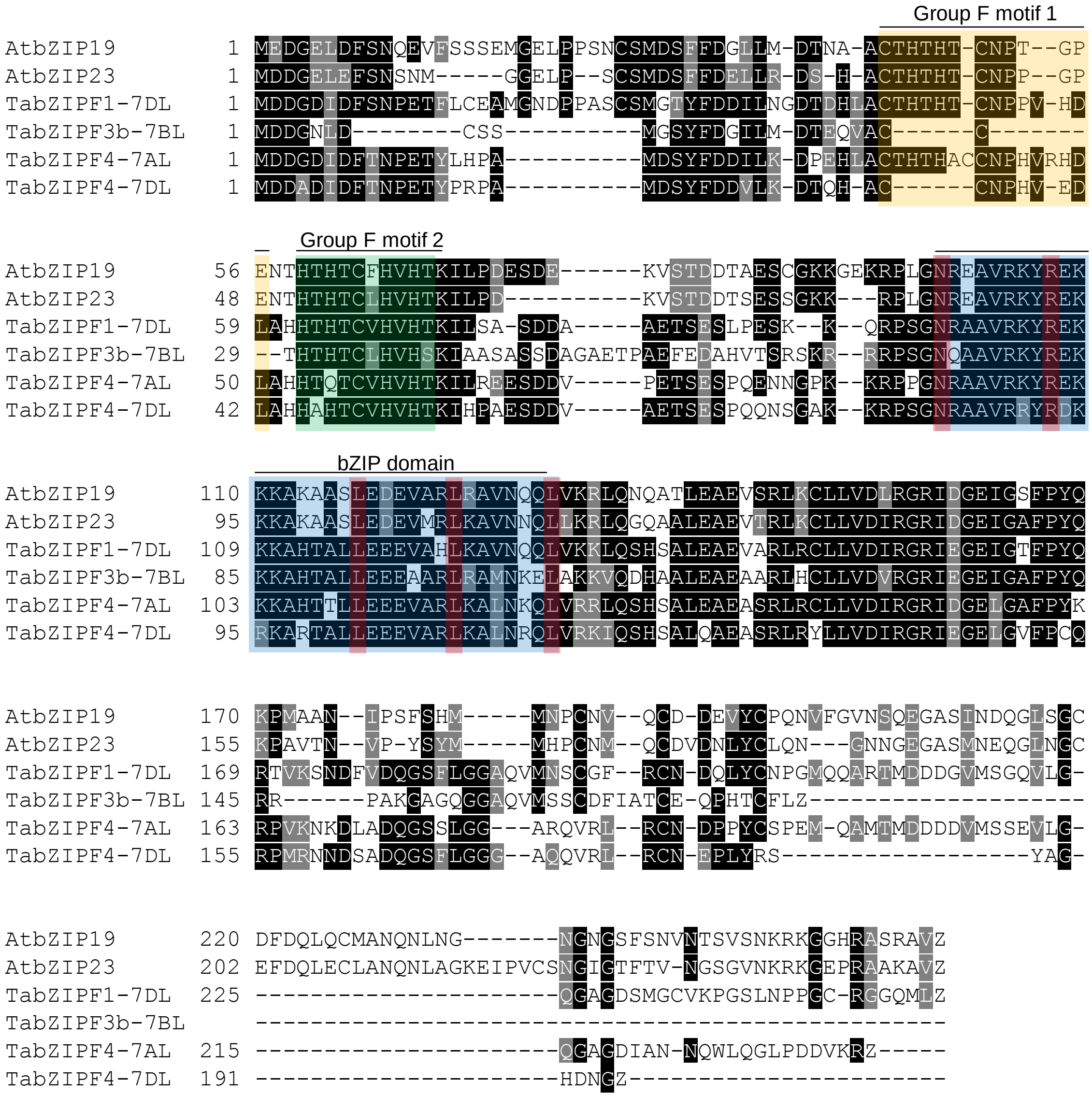

Figure S6. Multiple sequence alignment of cloned group F TabZIPs, AtbZIP19 and AtbZIP23. The Muscle algorithm (Edgar, 2004) was used for the alignment of sequences and the BoxShade software was used to generate this figure. The two group F motifs are labelled with lines above the alignment and shaded in yellow and green in consensus with the sequence prediction by Jakoby et al., (2002); motif 1 C[ST]HTH[ST]CNP[PT]GPE, motif 2 H[ST]HTC[FL]H[AV]HT. The bZIP domain common to all bZIP groups is also labelled and shaded in blue, the consensus of which is N-x_7-R/K-x_9-L-x_6-L-x_6-L, shown in red.
